# The Thymic Microenvironment Shapes Age‐Related Qualitative Changes in the TCR Repertoire

**DOI:** 10.1111/acel.70675

**Published:** 2026-08-20

**Authors:** Jasmine Rowell, Diana C. Yanez, Susan Ross, Ching‐In Lau, Eden Zhang, Amir Monfared, Oscar A. Peña, Benny Chain, Tessa Crompton

**Affiliations:** ^1^ UCL Great Ormond Street Institute of Child Health London UK; ^2^ School of Biochemistry, University of Bristol Bristol UK; ^3^ Division of Infection and Immunity University College London London UK

## Abstract

Ageing impairs immune function, increasing susceptibility to infection, autoimmunity and inflammation. The thymus undergoes involution during childhood, and thymocyte and thymic epithelial cell (TEC) numbers decline. Given that adaptive immunity depends on T‐cells recognising diverse antigens via their unique T‐cell receptor (TCR), the precise age‐related changes in TCR repertoire composition are key to understand immunity. To investigate the influence of age on the thymic TCR repertoire we sequenced TCRα and TCRβ transcripts from developmentally defined thymocyte populations from 4‐week, 12‐month and 18‐month‐old mice, and from thymocytes recovering from hydrocortisone‐treatment, and athymic recipients of RAG1‐deficient thymus transplants. The 18‐month thymus selected TCR repertoires with distinctive patterns of VxJ and CDR3 k‐mer usage compared to young thymus, indicating it had a qualitatively different repertoire with different specificity. It also selected a less diverse TCRβ repertoire with more expanded clones than young thymus. On recovery from hydrocortisone‐treatment, both 4‐week and 12‐month thymus showed bias towards foetal‐like 3′TRAVx5′TRAJ rearrangements, but 12‐month thymus also produced less diverse and evenly distributed TCRβ repertoires, with increased expansion of TCRβ clones at the CD4^−^CD8^−^CD25^+^CD44^−^intracellularTCRβ^+^ stage, increased TRBJ‐1‐cluster usage and distinctive patterns of combinatorial VxJ usage. Transplantation of athymic nude recipients with old RAG1‐deficient thymus generated less diverse, less evenly distributed TCR repertoires with different combinatorial VxJ usage than those generated by young transplants. Taken together our experiments show that the old thymus generates qualitatively different TCR repertoires than young thymus and the non‐lymphoid compartment of the thymus contributes strongly to these changes and to age‐associated decline in TCR repertoire diversity.

## Graphical abstract

Ageing profoundly alters the thymic microenvironment, but its impact on T‐cell receptor (TCR) repertoire selection remains unclear. By sequencing TCRαβ repertoires from young and aged thymus, hydrocortisone‐depleted thymus and RAG1‐deficient thymus transplantation models, we show that the aged thymic microenvironment drives qualitative changes in TCR selection and reduces repertoire diversity.

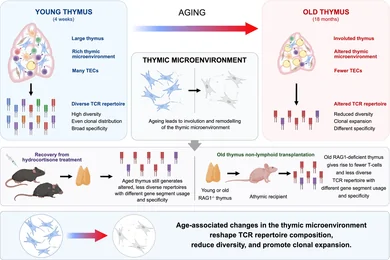

## 1 Introduction

Ageing compromises the normal functioning of the immune system, causing higher susceptibility to pathogens, lower vaccine responses and an increase in autoimmunity and inflammation (Weyand and Goronzy 2016; Oh et al. 2019; Conrad et al. 2023; Hester et al. 2022). The thymus is the sole lymphoid organ where T‐cell development occurs, and is made up of developing T‐cells (thymocytes) and stromal cells, including thymic epithelial cells (TEC) and fibroblasts, endothelial cells, macrophages and dendritic cells (Muro and Nitta 2025; Kadouri et al. 2020). In childhood, the thymus involutes and decreases in size in mice and humans, and the number of thymocytes and TEC decline (Linton and Dorshkind 2004; Palmer 2013; Wedemeyer et al. 2025). This is an evolutionary conserved process that reduces overall T‐cell output (Shanley et al. 2009). Despite this rapid involution, thymus function remains essential throughout the lifespan, as shown by the detrimental impact of thymectomy on immunity and health (Afifi et al. 2010; Prelog et al. 2009; Zlamy and Prelog 2009; Gudmundsdottir et al. 2018; Mengrelis et al. 2021; Kooshesh et al. 2023). In old age, the immune system is compromised by poor adaptive immune responses and increased self‐reactive T cells, ultimately weakening immunity to viral and bacterial infections and promoting inflammation and autoimmunity (Palmer 2013; Goronzy and Weyand 2005; Mitchell et al. 2010; Terekhova et al. 2025). Thymus dysfunction in old age may contribute to this decline, as peripheral T‐cell replacement and central tolerance may be impaired (Liang et al. 2022). In support of this, recent studies have shown that thymic health in adults, assessed by radiographic images, is linked to better health, lower all‐cause mortality, lower cardiovascular mortality, reduced lung cancer incidence and better responses to immunotherapy in cancer patients (Bernatz et al. 2026a, 2026b); whereas in mice restoration of thymic function in older animals has been shown to improve immune responses to infections (Morales‐Sánchez et al. 2025).

Adaptive immunity depends on the ability of T‐cells to recognise a broad range of antigens through their unique T‐cell receptor (TCR): a heterodimer composed of an α and a β chain, generated from somatic DNA gene segment recombination in the thymus. Adaptive immunity therefore relies upon a broad repertoire of TCRs with different specificities to potentially unknown pathogens, so that the immune system can respond to any new threat. Although next generation sequencing has advanced our understanding of the TCR repertoire, it is not practically possible to sequence all TCRs present in an individual, so measurements of TCR repertoire diversity rely on sampling of the repertoire, and determination of its richness (the number of unique TCR sequences present) and its evenness (the clonal abundance: how evenly the different TCRs are distributed) within the sample, by calculation of diversity and distribution indices taken from Ecology and Economics (Laydon et al. 2015; Shannon and Weaver 1949; Ceriani and Verme 2012; Izraelson et al. 2018; Mika et al. 2025).

T‐cell development begins when haematopoietic progenitor cells migrate from the bone marrow to the thymus through the bloodstream. Ageing strongly affects the ability of HSC to differentiate and proliferate, and transplanted bone marrow cells or HSC from older mice do not produce T‐cells efficiently in younger recipients (Hirokawa et al. 1986; Tyan 1977). Early thymocytes lack CD4 or CD8 expression and are termed double negative (DN) cells. DN cells undergo TCRβ gene rearrangement to generate the CD4^+^CD8^+^ double positive (DP) population, via an immature single positive (ISP) intermediate. DP cells then undergo TCRα gene rearrangement and repertoire selection to differentiate into single‐positive (SP) either CD4^+^CD8^−^ (SP4) or CD8^+^CD4^−^(SP8) cells, which emigrate from the thymus to the periphery, giving rise to naïve T‐cells. At the developmental transition from DP to SP cell, positive selection determines MHC‐restriction by allowing only those thymocytes that bear TCR which interact with self‐MHC+peptide with appropriate affinity/avidity to survive and differentiate, while also ensuring that SP4 and SP8 populations express TCR appropriately restricted by MHCII and MHCI, respectively (Littman 2016). The process is initiated through low level TCR‐signalling which leads to cell surface TCR/CD3 upregulation, so that the CD3^−/lo^DP population can be regarded as pre‐positive selection, whereas the CD3^+/hi^ DP population has initiated repertoire selection and contains cells that will become either SP4 or SP8. TEC provide essential signals that guide T‐cell development and shape the TCR repertoire, while lymphocyte‐stromal crosstalk is essential for full maturation of the thymic microenvironment (Muro and Nitta 2025).

Cortical (c) TEC mediate T‐cell lineage commitment and positive selection, while medullary (m) TEC orchestrate later stages of T‐cell development, such as negative selection of self‐reactive clones through presentation of Tissue Restricted Antigens (TRA), usually at the SP stage, and regulatory T‐cell (Treg) maturation (Kadouri et al. 2020). In mice, the frequency and regeneration of TEC and TRA expression by TEC decrease with age, limiting thymus function (Griffith et al. 2012; Baran‐Gale et al. 2020; Kousa et al. 2024; Lancaster et al. 2022; Venables et al. 2019).

As T‐cells develop, the TCR β and α gene loci undergo sequential rearrangements that establish a highly diverse TCR repertoire (Schatz and Swanson 2011). Successful rearrangement of the TCRβ locus and expression of the pre‐TCR are essential for directing thymocytes towards the αβ T‐cell lineage and driving their transition from the CD25^+^ DN3 stage to the DP stage and involves immune synapse formation and MHC‐interactions (Allam et al. 2021). Allelic exclusion at the TCRβ locus reduces simultaneous rearrangement and expression of both alleles and ensures that each developing thymocyte expresses only a single TCRβ chain. In contrast, the TCRα locus rearranges biallelically, with VJ recombination occurring on both chromosomes (Carico and Krangel 2015; Carico et al. 2017; Genolet et al. 2012). The TCRα locus is capable of multiple successive rearrangements on each allele, proceeding in an orderly fashion: recombination typically starts at the proximal gene segments (3′ TRAV with 5′ TRAJ) and progressively extends to more distal combinations (5′ TRAV with 3′ TRAJ) as development proceeds (Carico and Krangel 2015; Carico et al. 2017; Park et al. 2020). Therefore, when the thymus is recovering from depletion, such as following hydrocortisone treatment, there is an enrichment of proximal 3′ TRAV‐5′ TRAJ rearrangements (Rowell et al. 2024).

Human TCR repertoire diversity decreases with age (Weng 2023). Although this has been shown by several studies that have investigated the peripheral repertoire in ageing humans, it is not practical to determine the impact of ageing on the thymic TCR repertoire in healthy people. Therefore it is unclear whether the decline in peripheral TCR repertoire diversity is the result of the decline in thymus function or because of peripheral clonotypic expansion and exhaustion. Longitudinal studies demonstrated a decrease in TCRβ diversity in peripheral T‐cells circulating in blood from older human donors when compared to younger donors, with CD8 repertoires showing increases in clonal expansion (Britanova et al. 2014; Yoshida et al. 2017; Sun et al. 2022; Fang et al. 2025; Britanova et al. 2016). The proportion of naïve T‐cells decreases with age, and expansion of large naïve clones has been observed in elderly individuals which skewed the distribution of the naïve T‐cell repertoire, so that elderly naïve repertoires show a modest decline in richness (Qi et al. 2014; Lin et al. 2016; Alpert et al. 2019). Single cell RNA sequencing also demonstrated lower peripheral TCRαβ diversity in older adults, and reduced public TCRαβ clonotypes (van de Sandt et al. 2023). Furthermore, in aged TCRβ repertoires, a reduction was observed in both CDR3 length and the number of N nucleotides added (Egorov et al. 2018).

In mice, the proportion of naïve peripheral T‐cells and TCR repertoire diversity have also been shown to decline with age, when 23‐month‐old mice were compared to 3‐month‐old mice (Izraelson et al. 2018). Another study compared peripheral T‐cell repertoires from 3 month‐old and 12‐month‐old mice and found that MHC‐restriction had a greater impact on the repertoire than peripheral differentiation or age (Mark et al. 2022).

Here we investigated the impact of ageing on the TCR repertoire generated in the murine thymus, comparing 4‐week, 12‐month and 18‐month‐old thymocyte populations. Our findings show that ageing reduces the diversity and increases the clonality of the TCR repertoire generated in the thymus, and most importantly we show that ageing also leads to qualitative changes in the specificity of the repertoire selected.

## 2 Results

### 2.1 TCR Repertoires From the Aged Thymus Are Distinct From Young Adult Thymus in Diversity, Distribution, CDR3 Length and CDR3 Specificity

**FIGURE 1:**
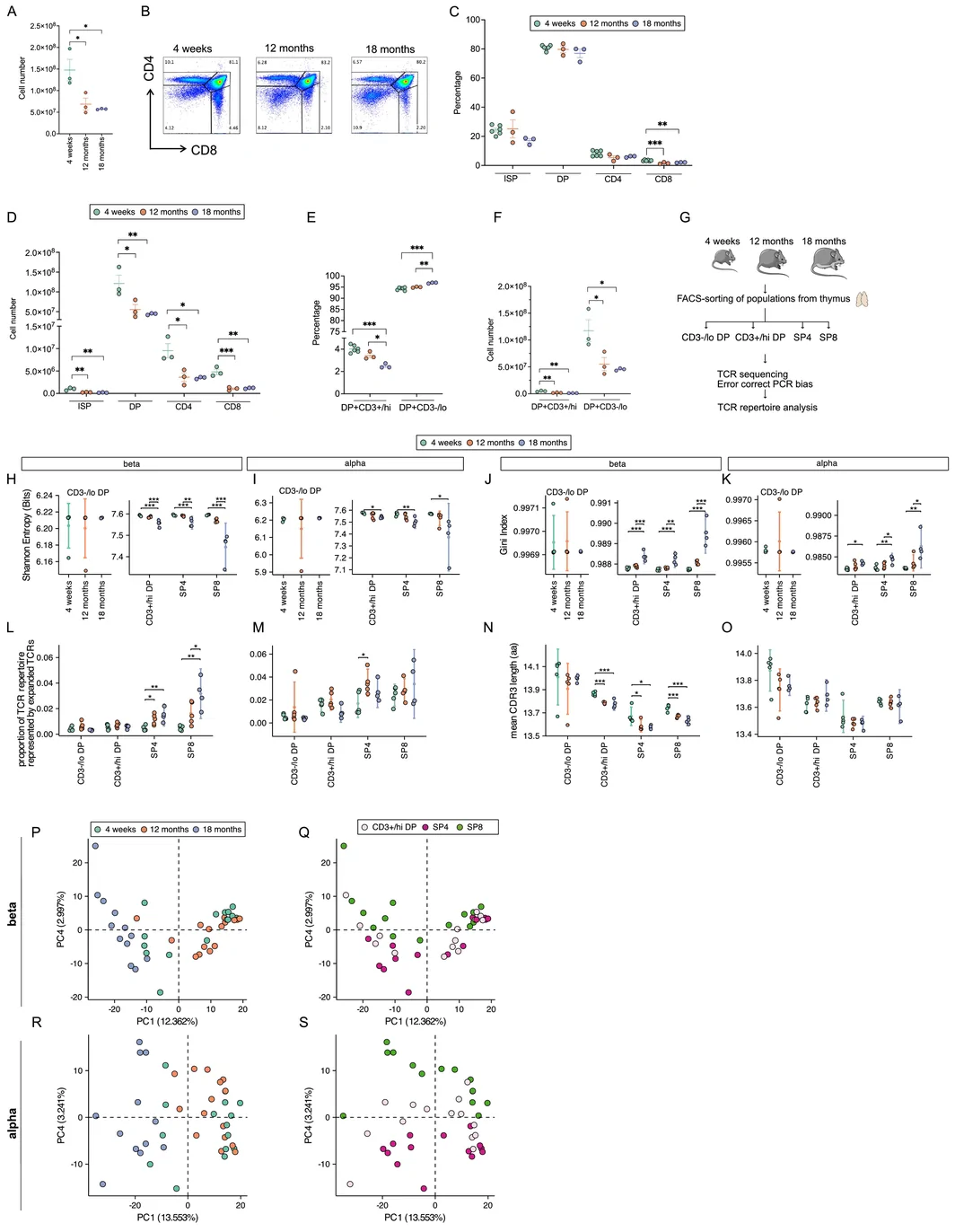
Thymus phenotype and TCR repertoires from aged and young adult mice. (A–F) Flow cytometry of thymus of 4‐week, 12‐month and 18‐month C57BL/6 mice from our colony (*n* = 3–6). (H–S) TCR α & β‐chain repertoires were sequenced from FACS‐sorted CD3^−/lo^CD4^+^CD8^+^ (CD3^−/lo^DP), CD3^+/hi^CD4^+^CD8^+^ (CD3^+/hi^DP), CD4^+^CD8^−^CD3^+^ (SP4) and CD4^−^CD8^+^CD3^+^ (SP8) thymocyte populations from 4‐weeks (green circles; *n* = 5), 12‐months (orange circles; *n* = 5) and 18‐months (purple circles; *n* = 4) C57BL/6 thymus. (A) Plot shows mean ± SEM cell number of thymus at 4‐weeks (green), 12‐months (orange) and 18‐months (purple) (*n* = 3–6). (B) Representative Flow cytometry analysis of thymocyte populations showing expression of CD4 and CD8 at 4‐weeks, 12‐months and 18‐months. (C, D) Plots show mean ± SEM percentage (C) and cell number (D) of different thymocyte populations. (E, F) Plots show mean ± SEM percentage (E) and cell number (F) of DP^+^CD3^+^ and DP^+^CD3^−^ populations at 4‐weeks, 12‐months and 18‐months. (G) Schematic showing design of experiments. 4‐weeks, 12‐months and 18‐months murine thymocytes were FACS‐sorted and TCR sequenced, with a protocol where each individual TCR mRNA was tagged with a UMI. (H, I) The Shannon entropies for the β‐chain (H) and α‐chain (I) repertoires of 4‐weeks, 12‐months and 18‐months in CD3^−/lo^DP (left plot) and CD3^+/hi^DP, SP4 and SP8 (right plot) populations, where each point represents an individual mouse. Each repertoire was subsampled to 500 TCRs 1000 times (for CD3^−/lo^DP) and to 2000 TCRs 1000 times (CD3^+/hi^DP, SP4 and SP8) before calculating the Shannon entropy. (J, K) The Gini index for the β‐chain (J) and α‐chain (K) repertoires of 4‐weeks, 12‐months and 18‐months in CD3^−/lo^DP (left plot) and CD3^+/hi^DP, SP4 and SP8 (right plot) populations, where each point represents an individual mouse. Each repertoire was subsampled to 500 TCRs 1000 times (for CD3^−/lo^DP) and to 2000 TCRs 1000 (CD3^+/hi^DP, SP4 and SP8) before calculating the Gini index. (L–M) The proportion of the total repertoire accounted for by the expanded top 0.1% most abundant sequences (> 99.9th percentile) for β‐chain (L) and α‐chain (M) for 4‐weeks, 12‐months and 18‐months in CD3^−/lo^DP, CD3^+/hi^DP, SP4 and SP8 populations, where each point represents an individual mouse. (N, O) The mean of β‐chain (N) and α‐chain (O) CDR3 length (number of amino acids) for 4‐weeks, 12‐months and 18‐months in CD3^−/lo^DP, CD3^+/hi^DP, SP4 and SP8 populations. (P–S) PCA biplot of β‐chain (P, Q) and ⍺‐chain (R, S) CDR3 amino acid triplet frequency distributions for 4‐weeks, 12‐months and 18‐months in CD3^+/hi^DP, SP4 and SP8 populations. (P, R) 4‐weeks, 12‐months and 18‐months samples are coloured in light green, orange and purple respectively, while in (Q, S) CD3^+/hi^DP, SP4 and SP8 populations are coloured light pink, dark pink and dark green respectively. (H–S) Dotplots show mean ± c.i. Statistical comparisons were performed within each cell‐type and were carried out by one‐way ANOVA followed by Tukey post hoc tests (significant *p* values shown): ***p < 0.001; **p < 0.01; *p < 0.05.

Before comparison of the TCR repertoires generated in the young (4‐week) and ageing (12‐month and 18‐month) C57BL/6 thymus, we characterised thymocyte populations in a small group of these mice from our colony by flow cytometry, to confirm known age‐related changes in thymocyte subset distribution (Figure 1A–F and Figure S1A–J). As expected, the 4‐week thymus was larger than both 12‐month and 18‐month thymus (Figure 1A). It contained a greater proportion of SP8, and more cells in ISP, DP, SP4 and SP8 populations, whereas we detected no differences in cell number in these populations between 12‐month and 18‐month thymus (Figure 1B–D and Figure S1A,B). Within the SP4 populations, the proportion of CD25^+^Foxp3^+^ cells (Treg) was more varied in the 12‐month and 18‐month thymus compared to 4‐week, and the percentage of Nrp1^+^ cells was higher in 12‐month and 18‐month thymic Treg populations (Figure S1C,D). The aged thymus contained fewer DN1, DN3 and DN4 cells than the young thymus (Figure S1E–G). The proportion of DN1 cells that expressed cell surface c‐kit was higher in the 12‐month thymus than in 4‐week or 18‐month, whereas there was no difference in c‐kit expression within the DN2 populations, and overall, the number of early T‐lineage progenitors (ETP) was lower in 12‐month and 18‐month thymus than in 4‐week (Figure S1H–J).

Within the DP population, the 4‐week‐old (young adult) and 12‐month thymus both contained a greater proportion of CD3^+/hi^DP cells and lower proportion of CD3^−/lo^DP cells than the 18‐month thymus, and the number of CD3^+/hi^DP and CD3^−/lo^DP cells was higher in the young thymus than in both 12‐month and 18‐month thymus (Figure 1E,F). Given these differences within the DP population, we FACS‐sorted CD3^−/lo^DP, CD3^+/hi^DP, SP4 (CD4^+^CD8^−^CD3^+^) and SP8 (CD4^−^CD8^+^CD3^+^) populations from 4‐week‐old, 12‐month‐old and 18‐month‐old thymus, and sequenced their rearranged TCR transcripts. We used a TCR sequencing and analysis pipeline that includes a single‐strand DNA ligation step to tag each molecule of TCR α and β‐chain mRNA with a unique molecular identifier (UMI), enabling correction for PCR bias and sequencing error in analysis (Oakes et al. 2017; Uddin et al. 2019; Thomas et al. 2013) (Figure 1G).

To compare the diversity and distribution of the repertoires generated in the different ages of thymus, we calculated the Shannon entropy, as a standard index of diversity and richness, which takes into account both the number of unique clonotypes and the frequencies of each clonotype (Shannon and Weaver 1949); and the Gini index, which quantifies the evenness of the distribution (Ceriani and Verme 2012). We found no significant differences in Shannon entropy of TCRβ and TCRα repertoires calculated from sampling the CD3^−/lo^DP repertoires sequenced from the different ages of mice (Figure 1H,I).

For CD3^+/hi^DP, SP4 and SP8 populations, the Shannon entropy was higher in the 4‐week repertoires than in the 18‐month repertoires, demonstrating a more diverse TCR repertoire in the younger adult thymocyte populations, and a clear impact of ageing on thymocyte TCR repertoires after initiation of repertoire selection (Figure 1H,I). The TCRβ and TCRα CD3^+/hi^DP, SP4 and SP8 repertoires in 18‐month thymus were also less evenly distributed (higher Gini index) in comparison to 4‐week repertoires (Figure 1J,K). To test if the reduction in diversity and evenness of distribution was the result of an increase in clonotypic expansion of abundant clones, we compared the proportion of the TCR repertoire represented by the top 0.1% most abundant TCR clones in each population (Figure 1L,M). In 12‐month and 18‐month SP4 β‐chain repertoires this was more than twice that of the young adult SP populations, whereas in 18‐month SP8 repertoires, the proportion of the TCRβ repertoire represented by the top 0.1% most abundant clones was more than three‐fold higher than in 4‐week repertoires (Figure 1L).

The complementary determining region 3 (CDR3) of the TCRβ chain, its most hypervariable region and the primary site of antigen recognition, is encoded by the combination of V‐D‐J gene segments, with additional diversity generated by non‐template additions/deletions. Mean TCRβ CDR3 length was shorter in 18‐month and 12‐month CD3^+/hi^DP, SP4 and SP8 TCRβ repertoires than in young adult, but not different in α‐chain repertoires (Figure 1N,O).

Although the thymic repertoire is presumably selected on a set of self‐peptides which are likely to be similar between mice of identical genetic background, the huge potential diversity of the repertoire means that in practice, overlap (Jaccard index) of identical TCRs (CDR3) between mice is low and does not differ between ages (Figure S2A,B). To look in more depth at TCR specificity, CDR3 sequences can be broken down into small amino acid motifs made up of sequential amino acid triplets (k‐mers) (Thomas et al. 2013). These can provide insight into MHC+peptide specificity of the CDR3, and may recur and be encoded by different gene segment combinations together with non‐genomic sequence. We therefore calculated the distribution of all possible combinations of any three sequential amino acids (3‐mers) found within the CDR3 region of each TCR from each repertoire. To explore whether the TCR repertoires of the different ages of mice differed qualitatively, as well as showing reduced diversity, we then carried out Principal Component Analysis (PCA) on the k‐mer (3‐mer) distribution frequencies of CD3^+/hi^DP, SP4 and SP8 populations (Figure 1P–S). PCA is a multivariate statistical method, which can be used to cluster datasets according to variability in values of multiple variants to detect dominant patterns (represented by the principal components (PCs)), which may have biological significance (Ringnér 2008). Both TCRα and TCRβ repertoires segregated broadly by age on PC1, separating the 18 month repertoires from most 4‐week and 12‐months. For α‐chain, repertoires also separated by cell‐type (SP4, SP8 and DP) on PC4.

#### 2.1.1 Summary

Ageing reduces TCR repertoire diversity and evenness of distribution after positive selection, with increased clonotypic expansion of abundant clones. K‐mer analysis indicated that the specificity of the repertoire generated in the old thymus is qualitatively different from that in the young thymus.

### 2.2 The Old Thymus Generates TCR Repertoires That Are Qualitatively Different From Young Thymus Repertoires

**FIGURE 2:**
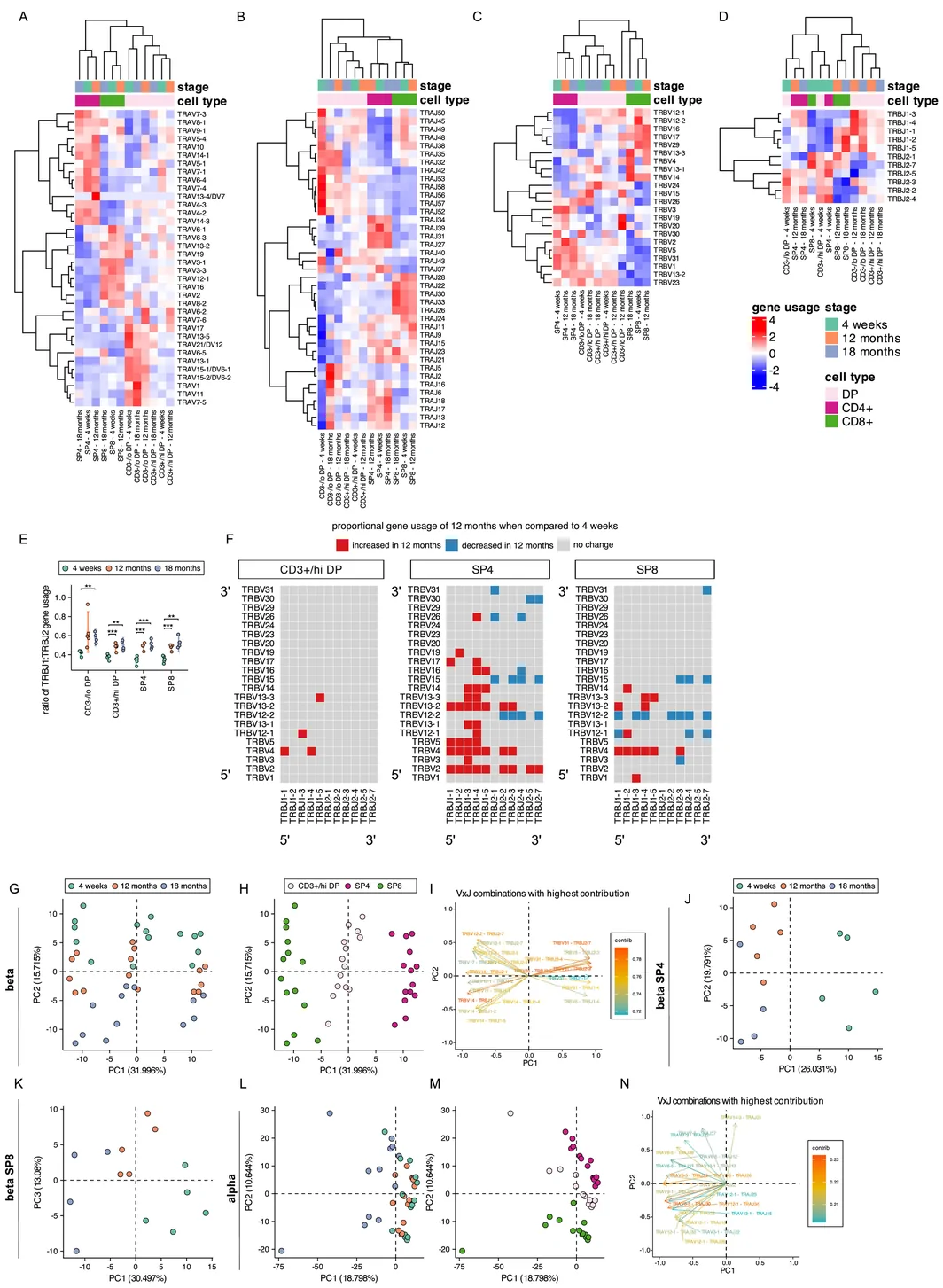
Age impacts the selection of VJ genes in thymic repertoires. TCR α and β‐chain repertoires were sequenced from FACS‐sorted CD3^−/lo^CD4^+^CD8^+^ (CD3^−/lo^DP), CD3^+/hi^CD4^+^CD8^+^ (CD3^+/hi^DP), CD4^+^CD8^−^CD3^+^ (SP4) and CD4^−^CD8^+^CD3^+^ (SP8) thymocyte populations from 4‐weeks (green circles; *n* = 5), 12‐months (orange circles; *n* = 5) and 18‐months (purple circles; *n* = 4) C57BL/6 thymus. (A–D) Heatmaps of proportional α‐chain variable (V) (A), α‐chain joining (J) (B), β‐chain V (C) and β‐chain J (D) gene usage of total TCRs for 4‐weeks, 12‐months and 18‐months in DP, SP4 and SP8 populations. Each column represents a mean of 4 or 5 mice and was clustered using Euclidian distance, while rows (genes) were clustered using Pearson correlation. (E) Ratio of TRBJ1:TRBJ2 cluster gene usage of total TCRs for 4‐weeks, 12‐months and 18‐months in DP, SP4 and SP8 populations. Each point represents an individual mouse. Dot plot shows mean ± c.i and statistical comparisons were carried out by one‐way ANOVA followed by Tukey post hoc tests (significant *p* values shown): ***p < 0.001; **p < 0.01. (F) Plots show comparison between β‐chain log normalised VxJ gene usage of unique TCRs for 12‐months compared to 4‐weeks in CD3^+/hi^DP, SP4 and SP8 populations. Red tiles signify increased usage of VxJ combinations in 12‐months (*p* < 0.05), while blue tiles signify decreased usage in 12‐months (*p* < 0.05) compared to 4‐weeks. Light grey tiles signify no change in gene usage (*p* > 0.05). Only combinations that were detected in at least 3 mice per group were compared. Statistical comparisons were carried out by unpaired Student's *t*‐test followed by FDR‐adjustment (5%, Benjamini‐Hochberg procedure) of *p* values. (G–N) PCA biplot of β‐chain (G–K) and ⍺‐chain (L–N) VxJ gene counts of total TCR sequences for 4‐weeks, 12‐months and 18‐months in CD3^+/hi^DP, SP4 and SP8 populations (G–I), SP4 alone (J) and SP8 alone (K). (G, J–L) 4‐weeks, 12‐months and 18‐months samples are coloured in light green, orange and purple respectively, while in (H, M) CD3^+/hi^DP, SP4 and SP8 populations are coloured light pink, dark pink and dark green, respectively. (I, N) Top 20 highest VxJ gene combinations that contribute to PC1 and PC2 of the PCA shown in (G, H) and (L, M), respectively.

We next compared gene segment usage in the four thymocyte populations between the three life‐stages of mice and visualised the differences in heatmaps (Figure 2A–D). For the α‐chain, samples clustered by cell‐type (SP4, SP8 and DP) for both TRAV and TRAJ, reflecting the influence of cell‐type (CD8^+^ or CD4^+^ cell, i.e., restricted by MHCI or MHCII) on gene segment usage (Figure 2A,B). The β‐chain V region usage also clustered by cell‐type, demonstrating the influence of cell‐type/MHC‐restriction on TRBV usage across adult life‐stages (Figure 2C). This is to be expected because the CDR1 and CDR2 loops in the β‐chain are encoded in the V gene segments and typically contact the MHC's conserved α‐helices, so they can determine MHC restriction during repertoire selection and TRBV usage has previously been shown to be influenced by cell‐type (Rowell et al. 2024; Wong et al. 2019; Camaglia et al. 2023). However, TRBJ usage failed to cluster by cell‐type, but showed a clear bias of older repertoires (12‐month and 18‐month) towards usage of TRBJ1‐cluster genes, whereas the 4‐week thymus showed increased usage of TRBJ2‐cluster genes (Figure 2D). Comparison of the ratio of TRBJ1 to TRBJ2 gene usage across the three ages of thymus showed a significantly higher ratio in the 18‐month CD3^−/lo^DP compared to the young CD3^−/lo^DP repertoires, whereas for CD3^+/hi^DP, SP4 and SP8 populations both 12‐month and 18‐month thymus had a significantly higher TRBJ1 to TRBJ2 ratio than young thymus (Figure 2E).

We next looked at the proportional usage of all possible TCRβ VxJ combinations in unique TCRβ sequences between 4‐week and 12‐month CD3^+/hi^DP, SP4 and SP8 thymocyte populations (Figure 2F). Four VxJ combinations were higher in the 12‐month CD3^+/hi^DP repertoires than in 4‐week, all of which used TRBJ1 cluster and 5′TRBV gene segments. The SP4 and SP8 12‐month repertoires showed many more differences, relative to 4‐week repertoires, with an overall bias towards increased proportional usage of 5′V to 5′J (TRBJ1‐cluster) rearrangements (Figure 2F). In contrast to the β‐chain repertoires, we detected no significant differences in the proportional usage of all possible TCRα VxJ combinations in unique TCRα sequences between 4‐week and 12‐month CD3^+/hi^DP, SP4 and SP8 thymocyte populations (Figure S2C).

To evaluate further the differences in TCRβ and TCRα proportional VxJ gene segment usage between young adult and aged thymocyte repertoires after initiation of positive selection, we carried out PCA of the β‐chain and α‐chain VxJ counts of CD3^+/hi^DP, SP4 and SP8 populations from the three groups of mice (Figure 2G–N). For the β‐chain, PCA separated the repertoires by cell‐type on PC1 and by age on PC2 (Figure 2G,H), but repertoires did not segregate by sex of mouse on any component. The VxJ combinations that contributed most strongly to PC1 and PC2 were distributed across all quadrants of the plot, so that the PCA highlighted different VxJ combinations that correspond to cell‐type (CD8^+^ cell or CD4^+^ cell, i.e., whether cell is restricted by MHCI or MHCII respectively) at different life‐stages (Figure 2I).

The Treg population in the aged thymus contains a greater proportion of cells that have recirculated into the thymus from the periphery (Yang et al. 2014; Santamaria et al. 2021), which therefore will have been subject to different selection pressures than T‐cells that have newly arisen in the thymus. It is possible that their TCRs in part account for the qualitative differences in the 4‐week and 18‐month repertoires observed (Figure 2G,H), where PC2 (separation by age) accounted for 15.7% of variability. Treg account for < 6% on average of the 18‐month SP4 population (Figure S1C), so their contribution to the 18‐month repertoires should not be sufficient to segregate the repertoires on PC2 by age, particularly given that SP8 and CD3^+/hi^DP repertoires were also segregated by age. However, to explore the variability between selected repertoires independent of the influence of cell‐type, we carried out separate PCA for each SP population (Figure 2J–K). For SP4, all 4‐week repertoires separated from all 18‐month and 12‐month repertoires on PC1 (26% variability); for SP8, all 4‐week repertoires separated from 18‐month repertoires on PC1 (30.5% variability). As both SP8 and SP4 β‐chain repertoires separated by age on PC1, the 18‐month thymus produces qualitatively different TCR repertoires than the 4‐week thymus, irrespective of the possible influence of recirculating Treg on the 18‐month SP4 repertoires.

PCA of the α‐chain VxJ counts separated repertoires by cell‐type on PC2 and broadly by age on PC1 (Figure 2L–N).

The fact that PCA of both CDR3 k‐mers and VxJ combinations segregates 18‐month β‐chain and α‐chain CD3^+/hi^DP, SP4 and SP8 repertoires from young repertoires (Figures 1P–S and 2G–N) indicates that repertoire selection in the old thymus has selected a different repertoire containing different TCRs with different specificity than in the young thymus.

#### 2.2.1 Summary

The TCR repertoire generated in the aged thymus is qualitatively different from that generated in the young thymus. Old thymus shows bias towards selection of different VxJ combinations from young adult thymus.

### 2.3 Ageing Reduces Impact of Cell‐Type (MHC‐Restriction) on Combinatorial VxJ Gene Usage

**FIGURE 3:**
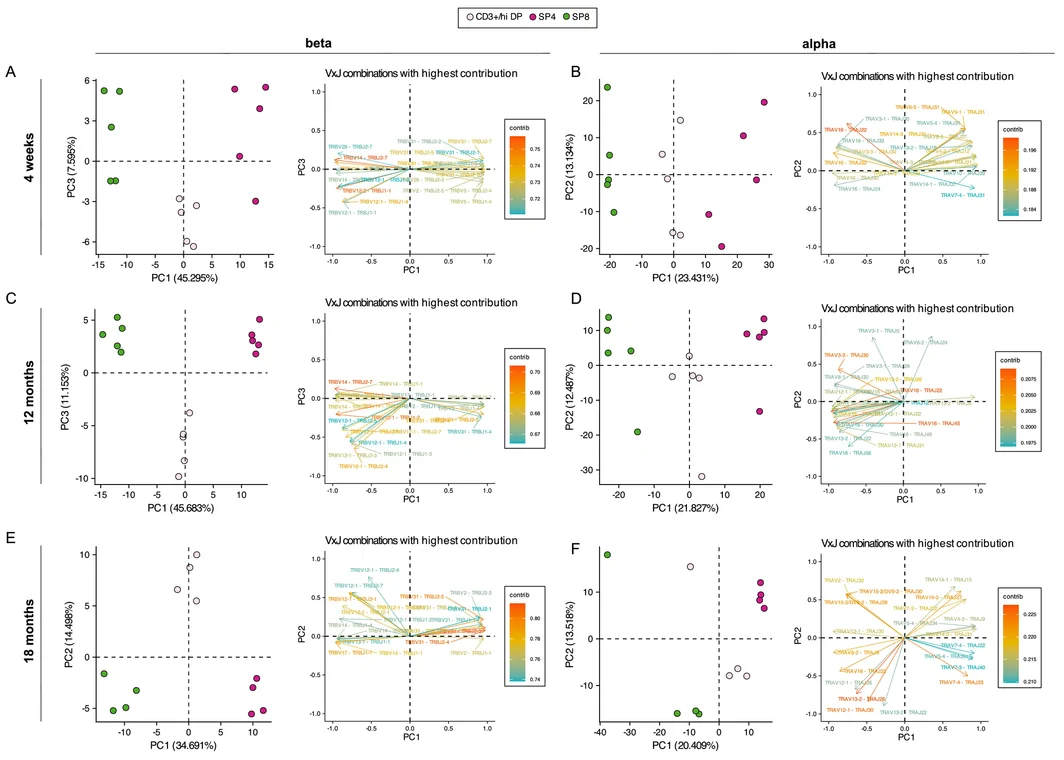
MHC‐restriction accounts for less variability in old TCR repertoires than in young TCR repertoires. TCR α and β‐chain repertoires were sequenced from FACS‐sorted CD3^+/hi^CD4^+^CD8^+^ (CD3^+/hi^DP), CD4^+^CD8^−^CD3^+^ (SP4) and CD4^−^CD8^+^CD3^+^ (SP8) thymocyte populations from 4‐weeks (green circles; *n* = 5), 12‐months (orange circles; *n* = 5) and 18‐months (purple circles; *n* = 4) C57BL/6 thymus. (A–F) PCA biplot of β‐chain (A, C, E) and α‐chain (B, D, F) VxJ gene counts of productive TCR sequences for 4‐weeks (A‐B), 12‐months (C, D), and 18‐months (E, F) in CD3^+/hi^DP (light pink), SP4 (dark pink) and SP8 (green) populations (left panel). Right panel shows the loading plot of the 20 VxJ gene combinations that contribute most to the PCA.

To investigate how ageing affects the influence of cell type (whether cells were restricted by MHCI or MHCII) on the thymic TCR repertoire we next carried out PCA for each life‐stage separately for the three thymocyte populations after initiation of repertoire selection (CD3^+/hi^DP, SP4 and SP8), using combinatorial VxJ counts as input (Figure 3A–F). For the 4‐week thymus, PCA of β‐chain combinatorial VxJ counts separated all repertoires by MHC‐restriction on PC1 (~45.3% variability), with CD3^+/hi^DP repertoires falling around the axis. Although most SP repertoires irrespective of cell‐lineage (SP4 or SP8) scored higher on PC3 (~7.6% variability) than CD3^+/hi^DP repertoires, SP repertoires did not fully segregate from CD3^+/hi^DP repertoires (Figure 3A). For the 12‐month and 18‐month thymus β‐chain, PCA again separated all repertoires by cell‐type on PC1 (~45.7% variability for 12‐month, ~34.7% variability for 18‐month) (Figure 3C,E). For 12‐month repertoires, PC3 (~11.2% variability) separated all SP repertoires from CD3^+/hi^DP repertoires. Of the 20 VxJ combinations that contributed most to the PC1 versus PC3 plot most fell on the negative (CD3^+/hi^DP) axis of PC3, suggesting that biases towards these combinations have been lost at the developmental transition from CD3^+/hi^DP to SP4 and SP8 (Figure 3C). For the 18‐month thymus PC2 (~15% variability) separated all SP repertoires from CD3^+/hi^DP repertoires (Figure 3E), and as observed for the 12‐month repertoires, nearly all of the 20 VxJ combinations that contributed most to the plot fell on the CD3^+/hi^DP axis, again suggesting that biases towards these combinations have been lost at the transition from CD3^+/hi^DP to SP cell in the older thymus.

Thus, in the 18‐month‐old thymus the influence of MHC‐restriction on β‐chain repertoires is reduced relative to 4‐weeks, while the CD3^+/hi^DP repertoires have become more distinct from SP repertoires, indicative of a proportional loss of some VxJ combinations at the transition from CD3^+/hi^DP to SP. This is consistent with fewer cells making the transition, or with the SP populations containing a greater proportion of cells that have recirculated back into the thymus from the periphery and been subject to different selection pressures, or with proportional loss of cells these TCR as a result of negative selection.

PCA of α‐chain combinatorial VxJ counts for the three life‐stages showed that in each case repertoires segregated by cell‐type on PC1, with a modest decline in the proportion of variability attributable to cell‐type (MHC restriction), from ~23.4% at 4‐weeks to ~20.4% at 18‐months; CD3^+/hi^DP repertoires did not segregate from SP repertoires on PC2 (Figure 3B,D,F).

#### 2.3.1 Summary

As the thymus ages the impact of MHC‐restriction on β‐chain repertoires declines, and the repertoire of the CD3^+/hi^DP population becomes more distinct from that of SP populations. The decline in combinatorial VxJ usage attributable to MHC‐restriction for the α‐chain is less than for the β‐chain.

### 2.4 Different Repertoire Generation of Young and Old Thymus During Recovery After Hydrocortisone Treatment

**FIGURE 4:**
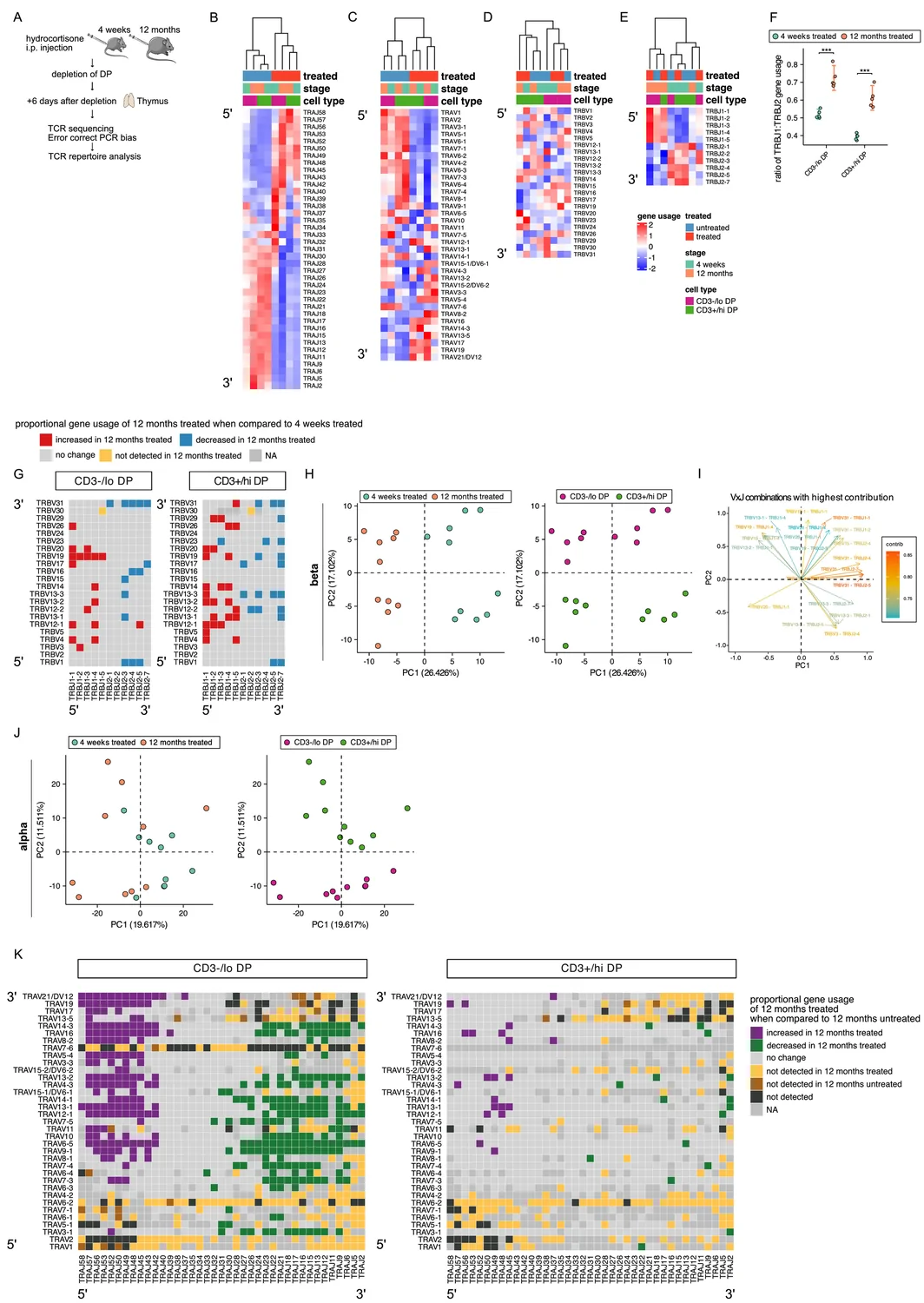
The recovering 4‐week and 12‐month thymus after hydrocortisone depletion showed enrichment for different VxJ combinations before and after positive selection. TCR α and β‐chain repertoires were sequenced from FACS‐sorted CD3^−/lo^CD4^+^CD8^+^ (CD3^−/lo^DP) and CD3^+/hi^CD4^+^CD8^+^ (CD3^+/hi^DP) thymocyte populations from 4‐week‐old treated with hydrocortisone on day 6 after treatment (green circles; *n* = 5) and 12‐month‐old treated with hydrocortisone on day 6 after treatment (orange circles; *n* = 5) C57BL/6 thymus. (A) Schematic showing design of experiments: 4‐week and 12‐month mice were treated with hydrocortisone resulting in the depletion of DP thymocytes. 6 days after treatment, the replenished CD3^−/lo^DP (CD3^−/lo^CD4^+^CD8^+^), and CD3^+/hi^DP (CD3^+/hi^CD4^+^CD8^+^) populations were FACS‐sorted and TCR sequenced. (B–E) Heatmaps of proportional α‐chain joining (J) (B), α‐chain variable (V) (C), β‐chain V (D) and β‐chain J (E) gene usage of total TCRs for 4‐weeks untreated and treated, along with 12‐months untreated and treated in DP populations. Each column represents a mean of 5 mice and was clustered using Euclidian distance, while rows (genes) were clustered using Pearson correlation. (F) Ratio of TRBJ1:TRBJ2 cluster gene usage of total TCRs for 4‐weeks and 12‐months treated in DP, SP4 and SP8 populations. Each point represents an individual mouse. Dot plot shows mean ± c.i. Statistical comparisons were performed within each cell‐type and carried out by one‐way ANOVA followed by Tukey post hoc tests (significant *p* values shown): ***p < 0.001. (G) Plots show comparison between β‐chain log normalised VxJ gene usage of total TCRs for 12‐months treated compared to 4‐weeks HC‐treated in CD3^−/lo^DP and CD3^+/hi^DP populations. Red tiles signify increased usage of VxJ combinations in 12‐months treated (*p* < 0.05), while blue tiles signify decreased usage in 12‐months treated (*p* < 0.05) compared to 4 weeks treated. Light grey tiles signify no change in gene usage (*p* > 0.05), yellow tiles signify VxJ combinations not detected in 12‐months treated, brown tiles signify VxJ combinations not detected in 4‐weeks treated and black tiles signify VxJ combinations not detected in both 12‐months treated and 4‐weeks treated. Dark grey tiles signify combinations that were not compared. Statistical comparisons were carried out by unpaired Student's *t*‐test followed by FDR‐adjustment (5%, Benjamini‐Hochberg procedure) of *p* values. Only combinations that were detected in at least 3 mice per group were compared. (H–J) PCA biplot of β‐chain (H, I) and ⍺‐chain (J) VxJ gene counts of unique TCR sequences for 4‐weeks treated and 12‐months treated in CD3^−/lo^DP and CD3^+/hi^DP populations. (H, J) 4‐weeks treated and 12‐months treated are coloured in light green and orange, respectively (left panel), while CD3^−/lo^DP and CD3^+/hi^DP populations are coloured dark pink and dark green, respectively (right panel). (I) Top 20 highest β‐chain VxJ gene combinations that contribute to PC1 and PC2 of the PCA in H. (K) Plots show comparison between ⍺‐chain log normalised VxJ gene usage of total TCRs for 12‐months HC‐treated compared to 12‐months untreated in CD3^−/lo^DP and CD3^+/hi^DP populations. Purple tiles signify increased usage of VxJ combinations in 12‐months treated (*p* < 0.05), while green tiles signify decreased usage in 12‐months treated (*p* < 0.05) compared to 12‐months untreated. Light grey tiles signify no change in gene usage (*p* > 0.05), yellow tiles signify VxJ combinations not detected in 12‐months treated, brown tiles signify VxJ combinations not detected in 12‐months untreated and black tiles signify VxJ combinations not detected in both 12‐months treated and 12‐months untreated. Dark grey tiles signify combinations that were not compared. Statistical comparisons were carried out by unpaired Student's *t*‐test followed by FDR‐adjustment (5%, Benjamini‐Hochberg procedure) of *p* values. Only combinations that were detected in at least three mice per group were compared.

As the steady‐state old thymus showed a decline in thymocyte number and TCR diversity, evenness of distribution, CDR3 length, and also selected distinct TCR during repertoire selection, we next disrupted thymus function to test the ability of the older thymus to generate a diverse TCR repertoire on recovery. Hydrocortisone (HC) treatment depletes the thymus of developing T‐cells, so that only cortisone‐resistant SP cells remain on day 2 after treatment (Rowbotham et al. 2009). It can therefore be useful to investigate the development and repertoire of thymocytes, as thymocyte populations recover in a synchronised wave of T‐cell development (Rowell et al. 2024; Solanki et al. 2020; Hager‐Theodorides et al. 2007). We treated 4‐week and 12‐month mice with HC and sequenced the TCRβ and TCRα repertoires from FACS‐sorted recovering CD3^−/lo^DP and CD3^+/hi^DP populations on day 6 after treatment (Figure 4A).

On Day 6 after treatment, the 12‐month thymus contained fewer cells than the 4‐week, with no change in the number of DN and CD3^+/hi^DP cells, but a decrease in the number of CD3^−/lo^DP in the 12‐month thymus compared to the 4‐week (Figure S3A–C). We previously showed that the 4‐week old thymus generates a more foetal‐like TCR repertoire during its recovery from HC‐depletion, with increased proportional use of 3′TRAV to 5′TRAJ combinations, and 5′TRBJ (Rowell et al. 2024). To visualise gene segment usage between HC‐treated and untreated 12‐month and 4‐week DP repertoires, we clustered repertoires by treatment, life‐stage and cell‐type in heatmaps (Figure 4B–E). For the TCR α‐chain, treatment (untreated versus HC‐treated) clustered all samples for both TRAJ and TRAV irrespective of cell‐type or life‐stage, with treatment increasing the use of 3′TRAV segments and 5′TRAJ segments at both life‐stages (Figure 4B,C). Thus, HC‐treatment has a greater influence over TRAV and TRAJ usage than either MHC‐restriction or age, and both the 12‐month and 4‐week thymus show a bias towards more foetal‐like TCRα gene segment usage when recovering from depletion, consistent with a rapid wave of synchronous development (Rowell et al. 2024).

For the TCRβ chain repertoires, TRBV usage clustered by cell‐type over age or treatment (Figure 4D), whereas TRBJ genes failed to cluster by cell‐type, treatment or age, but TRBJ1‐cluster genes showed enrichment in 12‐month populations, and the ratio of TRBJ1:TRBJ2 gene usage was higher compared to 4‐week populations (Figure 4E,F). Comparison of proportional combinatorial TRBJxTRBV usage between HC‐treated repertoires revealed increased usage of combinations that used TRBJ1‐cluster genes in 12‐month repertoires in comparison to 4‐week, and decreased proportional usage of combinations that use TRBJ2‐cluster genes (Figure 4G). PCA of frequency distributions of TRBJxTRBV combinations from HC‐treated repertoires separated 4‐week samples from 12‐month samples on PC1, whereas repertoires from CD3^−/lo^DP populations were separated from CD3^+/hi^DP populations on PC2 (Figure 4H,I). Thus, the recovering young and 12‐month thymus showed enrichment for distinct VxJ combinations both before and after entering positive selection. The biases towards TRBJ1 cluster gene usage observed in the older thymus and TRBJ2 cluster gene usage in the young thymus were maintained when the thymus recovered from HC‐depletion (Figure 4I).

PCA of frequency distributions of TRAJxTRAV combinations from HC‐treated repertoires separated CD3^−/lo^DP from CD3^+/hi^DP populations on PC2, but failed to separate 4‐week from 12‐week repertoires (Figure 4J), consistent with both HC‐treated 4‐week and 12‐week repertoires showing bias towards 3′TRAV and 5′TRAJ usage (Figure 4B,C). We found no significant differences in proportional usage of any TCRα VxJ combinations between HC‐treated 4‐week and 12‐month repertoires (Figure S3D). As the HC‐treated 4‐week thymus uses foetal‐like 3′TRAV and 5′TRAJ rearrangements during its recovery from HC‐treatment (Rowell et al. 2024), when thymocytes are expanding in a synchronised wave, we confirmed that the 12‐month thymus showed the same change in bias in TCRα rearrangements when recovering from HC‐treatment. Comparison of TRAVxTRAJ combinations between 12‐month HC‐treated and untreated CD3^−/lo^DP and CD3^+/hi^DP populations showed a proportional increase in 3′TRAV and 5′TRAJ combinations (> 150 combinations increased) and decrease in 5′TRAV and 3′TRAJ combinations (> 190 combinations decreased) in the HC‐treated 12‐month CD3^−/lo^DP population compared to HC‐untreated 12‐month control. This bias persisted but was less pronounced in the HC‐treated CD3^+/hi^DP population, which has initiated positive selection, suggesting that positive selection reduces bias in TCRα gene segment usage (Figure 4K).

#### 2.4.1 Summary

When recovering from HC‐treatment, 4‐week and 12‐month thymuses use different combinatorial β‐chain VxJ genes both before and after entering positive selection. Young thymuses use more TRBJ2‐cluster genes and old thymus uses more TRBJ1‐cluster genes. Both ages show bias towards 3′TRAV and 5′TRAJ usage.

### 2.5 Reduced TCR Repertoire Diversity and Distinct CDR3 k‐mer Usage in HC‐Depleted 12‐Month Compared to 4‐Week Thymus

**FIGURE 5:**
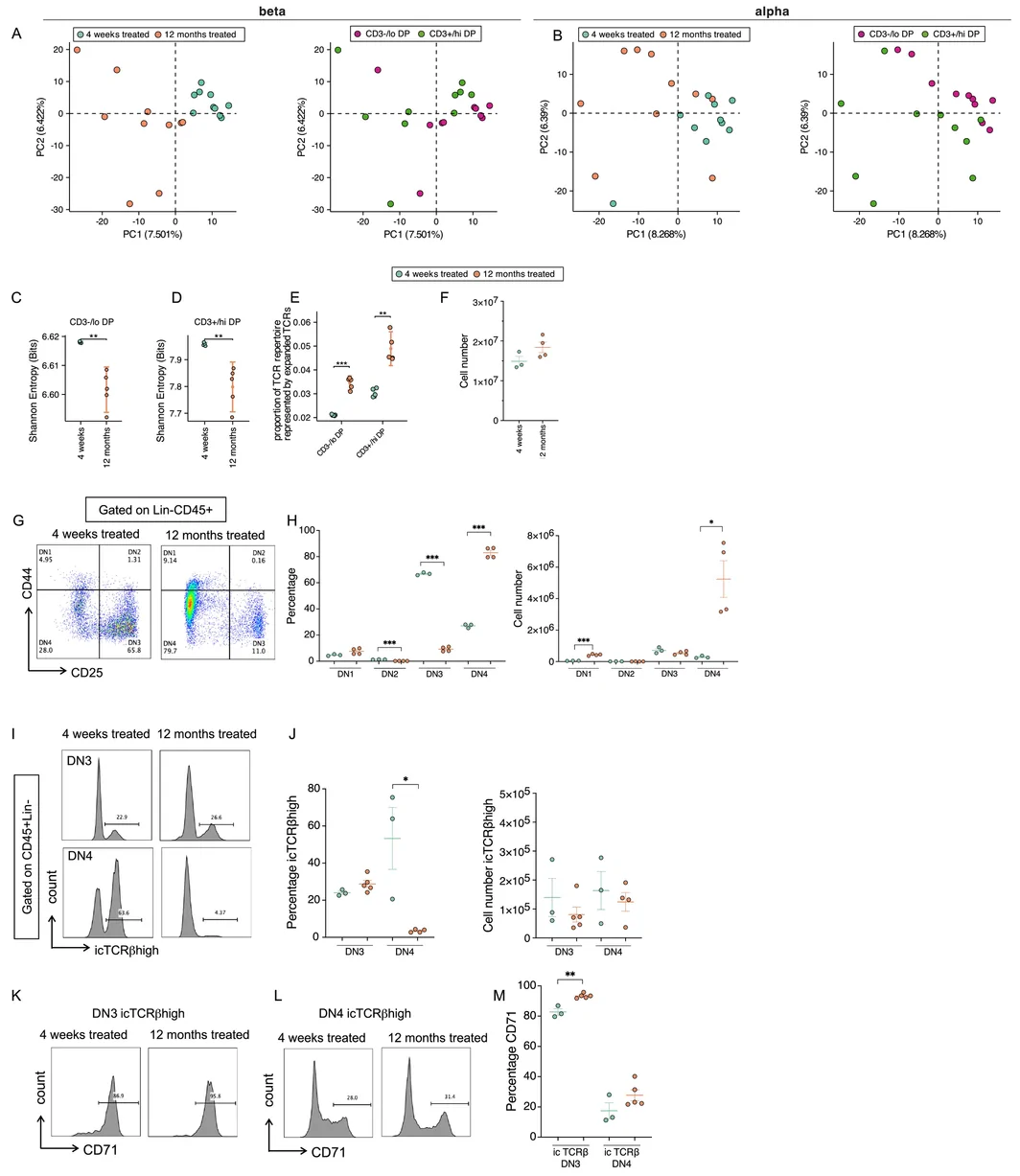
Aged thymus generated fundamental differences in CDR3 specificities and reduced repertoire diversity compared to young thymus following hydrocortisone depletion. Thymus was analysed after hydrocortisone (HC) treatment. (A–E) TCR α and β‐chain repertoires were sequenced from FACS‐sorted CD3^−/lo^CD4^+^CD8^+^ (CD3^−/lo^DP) and CD3^+/hi^CD4^+^CD8^+^ (CD3^+/hi^DP) thymocyte populations from 4‐week‐old treated with hydrocortisone on Day 6 after treatment (green; *n* = 5) and 12‐month‐old (orange; *n* = 5) C57BL/6 thymus on Day 6 after HC‐treatment. (F–M) Flow cytometry analysis of thymus on Day 3 after HC‐treatment of 4‐week (green; *n* = 3) and 12‐month (orange; *n* = 5) mice. (A–E) Day 6 after HC‐treatment. Dotplots show mean ± c.i (A, B) PCA biplot of β‐chain (A) and α‐chain (B) CDR3 amino acid triplet frequency distributions for 4‐weeks‐treated and 12‐months‐treated in CD3^−/lo^DP and CD3^+/hi^DP populations. 4‐weeks‐treated and 12‐months‐treated are coloured in light green and orange respectively (left), while CD3^−/lo^DP and CD3^+/hi^DP populations are coloured dark pink and dark green respectively (right). (C, D) Shannon entropies for the β‐chain TCR repertoires of 4‐weeks treated and 12‐months treated in CD3^−/lo^DP (C) and CD3^+/hi^DP (D) populations, where each point represents an individual mouse. Each repertoire was subsampled to 750 (C) or 3000 TCRs (D) 1000 times before calculating the Shannon entropy. (E) Proportion of the TCR β‐chain repertoire accounted for by the expanded top 1% most abundant sequences (> 99th percentile) for 4‐weeks treated and 12‐months treated in CD3^−/lo^DP and CD3^+/hi^DP populations, where each point represents an individual mouse. (F–L) Day 3 after HC‐treatment. Dot plots show mean ± SEM. (F) Dot plot shows cell number of thymus. (G) Expression of CD44 and CD25 markers gated on CD45^+^ and Lineage^−^ (CD3^−^, CD4^−^, CD8^−^, CD11b^−^, NK1.1^−^, CD19^−^ and Ter119^−^) to identify DN thymocyte populations. (H) Dot plots show percentage (left) and cell number (right) of DN populations. (I) Expression of icTCRβ gated on DN3 and DN4 populations. (J) Plots shows percentage (left) and cell number (right) of icTCRβ^high^ on DN3 and DN4 populations (mean ± SEM). (K, L) Histograms represent expression of CD71 in icTCRβ^high^ DN3 (K) and DN4 (L). (M) Dot plot shows percentage of CD71^+^ cells in different populations. Each point represents a thymus from an individual mouse. Statistical comparisons were performed within each cell‐type and were carried out by unpaired Student's *t*‐test or Welch's *t*‐test as appropriate: ***p < 0.001; **p < 0.01; *p < 0.05.

To investigate age‐dependent differences in the specificity of repertoires generated in the recovering HC‐depleted thymus, we carried out PCA of TCRβ and TCRα CDR3 k‐mer frequency distributions from the Day 6 repertoires. PCA clustered all HC‐treated TCRβ 4‐week repertoires and all but one TCRα 4‐week repertoires on the positive axis of PC1 of their respective PCA, whereas the 12‐month repertoires were more dispersed and the majority fell on the negative axis of PC1, demonstrating fundamental differences in the CDR3 specificities of TCR generated in older thymus following HC‐depletion (Figure 5A,B). We also compared the diversity and clonality of the repertoires in the recovering HC‐treated 4‐week and 12‐month thymus (Figure 5C–E, Figure S3E,F). Shannon entropy was lower in each HC‐treated population for 12‐month β‐chain and α‐chain repertoires compared to 4‐week (Figure 5C,D, Figure S3E) and the older repertoires showed an increase in the proportion of the repertoire represented by expanded clones (Figure 5E and Figure S3F), indicating that the older thymus generated a less evenly distributed and less diverse TCR repertoire on recovery from HC‐treatment.

To test if the 12‐month recovering thymus generated fewer productive TCRβ rearrangements at the DN stage, leading to a less diverse repertoire in the DP populations, we carried out flow cytometry analysis on Day 3 after HC‐treatment to investigate the dynamics of proliferation and differentiation of the DN cells that will give rise to the Day 6 DP populations in the two ages of mice (Figure 5F–M). On Day 3, the number of thymocytes recovered was not different between the two groups of mice (Figure 5F). The 12‐month thymus contained an increased number of DN1 and DN4 cells compared to 4‐week (Figure 5G,H), although there was no difference in the number of ETP (Figure S1K,L). The proportion of intracellular (ic)TCRβ^hi+^ cells in the DN3 population was similar in the 4‐week and 12‐month thymus, whereas the proportion of icTCRβ^hi+^ DN4 cells was lower in the old thymus (Figure 5I,J). After pre‐TCR signalling, DN3 (icTCRβ^hi+^) cells rapidly upregulate cell‐surface transferrin receptor (CD71) to undergo a burst of proliferation, so we compared cell‐surface CD71 expression on DN3 and DN4 cells between the 4‐week and 12‐month HC‐treated thymus. The 12‐month thymus contained a higher proportion of CD71^+^ cells within the DN3 population, indicating greater expansion of DN3 cells with successful TCRβ rearrangements in the older thymus compared to the young thymus (Figure 5K–M), consistent with the increased clonal expansion and reduced Shannon entropy observed in the 12‐month DP β‐chain repertoires on Day 6 (Figure 5C–E).

#### 2.5.1 Summary

The 12‐month thymus generates a less diverse and evenly distributed TCRβ and TCRα repertoire on recovery from HC‐treatment than the 4‐week thymus, with increased clonal expansion of TCRβ clones occurring early at the DN3 stage. K‐mer analysis demonstrates qualitative differences in CDR3 repertoires generated between young and old HC‐treated thymus.

### 2.6 Contribution of the Thymic Microenvironment to Age‐Related Decline in TCR Repertoire Diversity on Thymus Transplantation

To investigate the contribution of the non‐lymphoid compartment of the thymus to the age‐related decline in TCR repertoire diversity and distribution, we used a well‐established method of thymus transplantation (Chawda et al. 2023; Furmanski et al. 2013; Ross et al. 2018) to introduce thymus from young and old (12‐month) GFP^+^RAG1‐deficient donors into congenic athymic (nude) recipients. Nude mice have normal haematopoietic progenitor cells but no TEC, and therefore no T‐cells, because of mutation of the FoxN1 gene (Nehls et al. 1994); whereas in RAG1‐deficient thymus, thymocytes fail to develop beyond the DN3 stage, but stromal and TEC lineages are genetically normal, although their maturation may be affected by the absence of SP cells to provide crosstalk signals (Muro and Nitta 2025).

**FIGURE 6:**
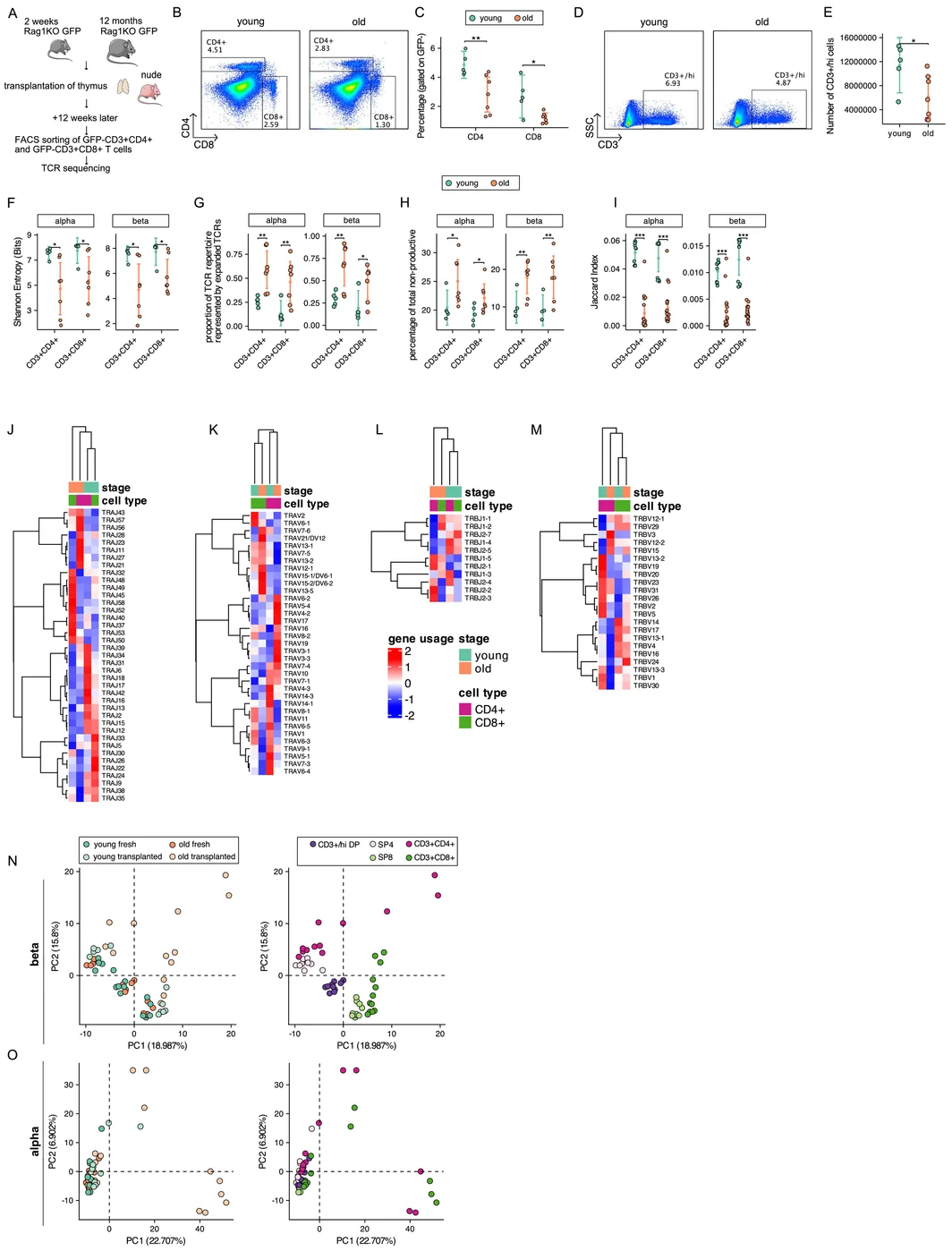
Old RAG1‐deficient thymus transplantation gives rise to a qualitatively different reconstituted TCR repertoire than young RAG1‐deficient thymus that is less diverse, less evenly distributed and has more non‐productive TCRs. Rag1^−/−^GFP^+^ 2‐week (young, green circles; *n* = 5) or 12‐month‐old (old, orange circles; *n* = 6) thymus was transplanted into recipient nude athymic C57BL/6 mice. After 12 weeks, TCR α and β‐chain repertoires were sequenced from FACS‐sorted GFP^−^CD3^+^CD4^+^CD8^−^ (CD3^+^CD4^+^) and GFP^−^CD3^+^CD4^−^CD8^+^ (CD3^+^CD8^+^) peripheral T‐cell populations. (A) Schematic of experimental design of thymus transplantation. Rag1^−/−^GFP^+^ 2‐week (young) or 12‐month‐old (old) thymus was transplanted into recipient nude athymic C57BL/6 mice. 12 weeks after transplant, peripheral CD3^+^CD4^+^CD8^−^ (CD3^+^CD4^+^) and CD3^+^CD4^−^CD8^+^ (CD3^+^CD8^+^) were FACS‐sorted and TCR sequenced. (B) Flow cytometry analysis of nude recipient mice 12 weeks after transplant of young and old thymus showing CD4 and CD8 expression. (C) Dot plot shows percentage of CD4^+^ and CD8^+^ populations from young and old transplanted. Each point represents an individual mouse. (D) Flow cytometry analysis of nude recipient mice 12 weeks after transplant of young and old thymus showing CD3 expression. (E) Dot plot shows percentage of CD3^+/hi^ populations from young and old transplanted. Each point represents an individual mouse. (F) The Shannon entropies for the α‐chain (left) and β‐chain (right) TCR repertoires of young and old transplanted in CD3^+^CD4^+^ and CD3^+^CD8^+^ populations, where each point represents an individual mouse. Each repertoire was subsampled to 4000 TCRs 1000 times before calculating the Shannon entropy. (G) The proportion of the total TCR α‐chain (left) and β‐chain (right) repertoires accounted for by the expanded top 1% most abundant sequences (> 99th percentile) for young and old transplanted, where each point represents an individual mouse. (H) The percentage of non‐productive α‐chain (left) and β‐chain (right) TCRs for young and old transplanted, where each point represents an individual mouse. (I) The intra life‐stage Jaccard Index of Similarity of α‐chain (left) and β‐chain (right) CDR3s for young and old transplanted in CD3^+^CD4^+^ and CD3^+^CD8^+^ populations. Each repertoire was subsampled to 3000 CDR3s 1000 times before calculating the Jaccard Index. (J–M) Heatmaps of proportional α‐chain joining (J) (J), α‐chain variable (V) (K), β‐chain J (L) and β‐chain V (M) gene usage of total TCRs for young and old transplanted in CD3^+^CD4^+^ and CD3^+^CD8^+^ populations. Each column represents a mean of 5–6 mice and was clustered using Euclidian distance, while rows (genes) were clustered using Pearson correlation. (N, O) PCA biplots of β‐chain (N) and α‐chain (O) VxJ gene counts of productive TCR sequences for CD3^+^CD4^+^ and CD3^+^CD8^+^ repertoires from young and old transplanted mice and fresh CD3^+/hi^DP, SP4 and SP8 repertoires from 4‐weeks (young) and 12‐months (old). Young‐fresh, old‐fresh, young‐transplanted and old‐transplanted repertoires are coloured in green, orange, light green and light orange respectively (left panel), while CD3^+/hi^DP, SP4, SP8, CD3^+^CD4^+^ and CD3^+^CD8^+^ populations are coloured purple, light pink, light green, dark pink and dark green respectively (right panel). Dotplots show mean ± c.i. Statistical comparisons were performed within each cell‐type and were carried out by unpaired Student's *t*‐test or Welch's *t*‐test as appropriate: ***p < 0.001; **p < 0.01; *p < 0.05.

After 12 weeks we compared the TCRβ and TCRα transcripts in the peripheral GFP^−^CD4^+^CD8^−^CD3^+^ and GFP^−^CD4^−^CD8^+^CD3^+^ T‐cells that were generated (Figure 6A). Recipients that received old thymus tissue had a lower percentage of peripheral CD4^+^ and CD8^+^ T‐cells and fewer CD3^+^ cells overall than those that received young thymus (Figure 6B–E). TCRα and TCRβ repertoires in both CD4 and CD8 populations from recipients with old thymus tissue were less diverse (lower Shannon entropy), and a higher proportion of their repertoire was represented by expanded clones than those from recipients of young thymus (Figure 6F,G). Additionally, old thymus transplants led to repertoires with a higher percentage of non‐productive rearrangements than young (Figure 6H). Most non‐productive TCR rearrangements are believed to be eliminated by nonsense‐mediated decay (Weischenfeldt et al. 2008), although some non‐productive TCR transcripts have been shown to persist in mouse and human T‐cells (Britanova et al. 2016; Camaglia et al. 2023; Mahowald et al. 2011; Yanez et al. 2025). Old thymus donors generated CD4 and CD8 repertoires that shared less β‐chain and α‐chain CDR3 sequences with one another than young donors (Figure 6I).

Comparison of gene segment usage between the two groups of mice showed that only TRAV and TRBV segregated by cell‐type (CD4^+^ or CD8^+^ cell), whereas TRBJ and TRAJ gene usage segregated by age of donor thymus (Figure 6J–M). Bias towards TRBJ1 usage was not found in the recipients with old thymus transplants, indicating that the TRBJ1‐bias is dependent on the age of the lymphoid compartment.

#### 2.6.1 Summary

Transplantation of athymic mice with old RAG1‐deficient thymus (containing old TEC and stromal cells) gives rise to fewer T‐cells, with TCR repertoire that is less diverse and evenly distributed than the repertoire generated by transplantation of young RAG1‐deficient thymus. Old thymus transplantation leads to more non‐productive rearrangements. Repertoires from recipients of old‐thymus‐transplants share fewer CDR3 between individuals than those from recipients of young‐thymus‐transplants.

### 2.7 Age of Thymus Microenvironment Affects TCR Repertoire Generated on Transplantation Into Nude Recipients

To assess how the transplant repertoires differed from normal repertoires we carried out PCA of VxJ counts from the transplant repertoires together with young and 12‐month repertoires from fresh thymus (Figure 6N,O). For β‐chain, all repertoires from fresh thymus and from young‐thymus‐transplant recipients segregated by cell‐type (CD4^+^ or CD8^+^ cell). In contrast, repertoires from old‐thymus‐transplant recipients failed to segregate by cell‐type in the PC1 v PC2 plot and were highly dispersed (Figure 6N). For the α‐chain, PCA of VxJ counts failed to separate repertoires by cell‐type on either PC1 or PC2, but all fresh thymus repertoires, and all but one repertoire from a young‐thymus‐transplant recipient fell on the negative axis of PC1, whereas repertoires from old‐thymus‐transplant recipients were widely dispersed, with the majority scoring high on the positive axis of PC1 (Figure 6O). These analyses together with the reduced sharing of clonotypes between individuals therefore indicate that selection of the repertoires generated in the old‐thymus‐transplant recipients was very different from that generated by young thymus, with a weaker influence of cell‐type (CD4^+^ or CD8^+^ cell) on β‐chain proportional VxJ usage, indicative of dysregulated repertoire selection.

#### 2.7.1 Summary

Selection of repertoires generated in the old‐thymus‐transplant recipients was different from the selection of young‐transplant repertoires.

## 3 Discussion

Here we employed a bulk population‐based strategy to investigate the TCRβ and TCRα chain repertoires from developmentally defined thymocyte populations from young adult (4‐week) and old (12‐month and 18‐month) mice. Our study revealed many differences between young adult and 18‐month thymus in repertoire diversity, distribution, clonality and TCR gene segment usage, with 18‐month SP repertoires showing reduced diversity of the TCRβ repertoire, with reduced mean CDR3 length, and bias towards TRBJ1 gene cluster usage, compared to 4‐week repertoires. Importantly, our analyses also showed that age of mouse influences combinatorial VxJ and CDR3 k‐mer usage in thymocyte populations, indicating that ageing affects selection of the TCR repertoire and leads to qualitative changes in the TCR repertoires generated in the old thymus compared to young. This indicates that in old age the thymus produces T‐cells that express different TCR with different specificity than in the young. These qualitative changes in the thymic TCR repertoire in old age therefore provide a new explanation for age‐associated decline in immunity.

The increased frequencies of clonally expanded TCRs in aged SP thymocytes could in part reflect recirculation of T‐cells that were expanded in the periphery. However, we observed that the CD3^+^DP repertoires, in addition to SP repertoires, from the old thymus had a less evenly distributed repertoire than those of their young counterparts, suggesting that clonal expansion was increased during T‐cell development in the old thymus. Likewise, we observed more clonal expansions in DP thymocytes recovering from HC‐treatment than in young counterparts.

To investigate how different thymus compartments contribute to age‐related changes in the TCR repertoire, we carried out cell transfer experiments. When transplanted into congenic nude recipients, 12‐month RAG1‐deficient thymus tissue generated a less diverse and rich TCR repertoire with more clonal expansions and more non‐productive rearrangements than young transplants. Additionally, the repertoires generated by old thymus transplants showed bias towards different VxJ combinations than those generated by young, with more variation between individuals and less influence of cell type, together suggesting that TCR repertoire selection in old‐thymus‐transplant recipients was dysregulated. We did not observe an age‐related bias towards TRBJ1 usage in these transplant experiments, indicating that this bias is determined by the haematopoietic compartment of the thymus. However, the transplant experiments highlight the impact of the non‐lymphoid compartment of the thymus on age‐related decline in TCR diversity and changes in repertoire selection. As TEC are the only lineage of thymic cells absent from the nude recipients and are an essential component of the thymus microenvironment for T‐cell development, we believe that they are the most likely candidate cell type responsible for the age‐dependent changes in TCR repertoire observed in the transplant experiments. The interpretation of this experiment is complicated by the fact that in a 12‐month‐old RAG1‐deficient mouse, TEC and stromal cells would have been present for a year without crosstalk from thymocytes beyond the DN3 stage, and so their differentiation may have been impaired, as crosstalk between TEC and lymphocytes is essential for TEC differentiation (Muro and Nitta 2025). It is possible that the young RAG1‐deficient environment was still capable of responding to crosstalk signals from developing nude thymocytes, while the 12‐month RAG1‐deficient thymic environment contained fewer TEC/stromal cells capable of differentiating fully in response to such signals, and thus supporting TCR repertoire selection.

Overall, several differences between the old and young thymus could account for age‐related changes in the TCR repertoire generated, and it is likely that both the haematopoietic compartment and stromal compartment/TEC contribute. Some qualitative differences in the TCR repertoire may be the result of differences in gene rearrangement between young and old that may in turn reflect changes in chromatin accessibility or rate of differentiation, both of which influence combinatorial gene segment usage and which may change across the life‐course (Park et al. 2020; Rowell et al. 2024; Ndifon et al. 2012). As the thymus ages, both the frequency and regeneration of TEC decline (Griffith et al. 2012; Baran‐Gale et al. 2020; Kousa et al. 2024; Venables et al. 2019). The resulting reduction in TEC‐thymocyte interactions could lead to fewer thymocytes completing repertoire selection (reduction in diversity) and could bias selection towards thymocytes that have been rearranging for longer and/or towards thymocytes bearing TCR which provide optimal signalling for positive selection. Indeed, after positive selection, the old thymus generated repertoires with shorter β‐chain CDR3 than young thymus, consistent with a study showing that shorter CDR3 lengths (8–13 amino acids) are optimal for MHC‐binding, because longer CDR3 reduce CDR1/2 contacts with MHC (Lu et al. 2019). Additionally, TEC function declines with age, so there may be functional differences, such as in expression of AIRE, peptide processing and MHC+peptide expression by TEC which lead to qualitative changes in the TCR repertoire selected in the old thymus (Griffith et al. 2012; Lancaster et al. 2022).

Although the steady‐state 12‐month and 18‐month thymus were similar in cell number and subset distribution, the 18‐month repertoires showed greater clonotypic expansion and reduction in diversity and more changes in combinatorial VxJ and CDR3 k‐mer usage relative to 4‐week thymus than the 12‐month thymus. Indeed, although the 12‐month thymus is smaller and produces fewer T‐cells than the 4‐week, by many measures (diversity indices, k‐mer and VxJ usage) in steady‐state it produces a qualitatively similar repertoire. However, when depleted by HC‐treatment, the 12‐month thymus recovered less efficiently than the young thymus and generated a less diverse TCRβ repertoire in both the pre‐positive selection CD3^−/lo^DP population and the selecting CD3^+/hi^DP population, with increased expansion of TCRβ clones at the DN3 stage and increased TRBJ1 cluster gene segment usage. Importantly, the recovering 12‐month thymus used different β‐chain VxJ combinations after initiation of positive selection than the young adult thymus, again demonstrating qualitative differences in the TCR repertoire selected in the older thymus. These differences between 4‐week and 12‐month thymus became apparent when T‐cell development was perturbed, but were less evident in the repertoires of the steady‐state thymus. This has implications for human health, as treatment with steroids or chemotherapy will significantly deplete the thymus and peripheral T‐cell populations, and in older patients, recovery of TCR repertoire diversity may be limited and may lead to generation of a qualitatively different repertoire.

Our approach of bulk TCR sequencing from FACS‐sorted thymocyte populations had the advantages of scale (allowing the sequencing of tens of thousands of rearrangements from thousands of cells from 35 individual mice), at relatively low cost. However, it had the disadvantage that we were unable to investigate TCRα/β pairing in single cells. In future it will be important and interesting to expand this analysis using single cell RNA sequencing.

Taken together, our data highlight the importance of the thymus for determination of the TCR repertoire across the life‐course, and the impact of ageing of thymic repertoire diversity, clonality and specificity.

## 4 Materials and Methods

### 4.1 Mice

C57/BL6 mice were bred at University College London (UK). B6.Cg‐Rag1‐deficient ubiquitin‐GFP mice (Rag1tm1Mom Tg(UBC‐GFP)30Scha/J (013115)) and nude mice (B6.Cg‐Foxn1nu/J (000819)) both on C57BL/6 genetic background were purchased from Jackson. All mice were bred and maintained in specific pathogen‐free conditions at University College London (UK) where animal experiments were carried out under UK Home Office regulations, following ethical review at UCL. Mixed sex of 4‐week‐old, 12‐month‐old and 18‐month‐old C57BL/6 mice were used in all experiments. For hydrocortisone (HC) treatment, mice were injected intraperitoneally with 0.6 mg/g of body weight pure HC sodium phosphate (Sigma Aldrich, cat. no: BP188) in sterile PBS, and analysed after 3 and 6 days as described previously (Rowell et al. 2024). Subcutaneous thymus transplantation of entire GFP+Rag1‐deficient 2‐week and 12‐month thymus into nude mice were carried out as described previously (Chawda et al. 2023).

### 4.2 Flow Cytometry

Tissues were disaggregated through a 70 μm cell strainer (VWR) to obtain single cell suspensions in AIMV medium. Cell suspensions were stained as described (Lau et al. 2021), using directly conjugated antibodies supplied by Biolegend or eBioscience (Table S1). Samples were acquired on a Cytoflex cytometer (Beckman) or LSR II (BD) and analysed in FlowJo 10.4.2 (Tree Star). Live cells were gated according to FSC/SSC profiles. For intracellular TCRβ staining, cells were fixed after surface staining and permeabilized using the Intracellular Fixation and Permeabilization Buffer Set (eBioscience San Diego, CA). Intracellular staining for Foxp3 was carried out as described (Papaioannou et al. 2019).

### 4.3 Fluorescence Activated Cell Sorting

Cell suspensions were stained and sorted on a BD FACS Aria III, as described (Rowell et al. 2024) for SP4 (CD4^+^CD8^−^CD3^+^), SP8 (CD4^−^CD8^+^CD3^+^) CD3^−/lo^DP (CD4^+^CD8^+^CD3^−/lo^) and CD3^+/hi^DP (CD4^+^CD8^+^CD3^+/hi^), as shown in Figure 1B and Figure S1A,B.

### 4.4 RNA Extraction, TCR Amplification and Sequencing

RNA was extracted as described (Rowell et al. 2024). We then used the protocol for TCR amplification and sequencing described in (Oakes et al. 2017; Uddin et al. 2019), with some minor modifications for mouse samples as described (Rowell et al. 2024).

### 4.5 Sequencing

Libraries were sequenced on the NextSeq using the NextSeq 500/550 Mid Output Kit v2.5 (300 Cycles) (Illumina, cat. no: 20024905) at UCL genomics.

### 4.6 Error Correction and Outputting

The NextSeq outputs files in the format named binary based call (.bcl) which were converted into FASTQ files using bcl2fastq for downstream processing using a pipeline of scripts described previously (Oakes et al. 2017; Thomas et al. 2013): *Decombinator_v3.1* (available at: https://github.com/innate2adaptive/Decombinator/) in Python 2.7. This pipeline identifies the errors introduced by PCR amplification using the UMI attached during the ligation step and thus produces an output of a corrected abundance of the TCR in the sample along with determining V, J and CDR3 regions for each TCR.

### 4.7 Downstream Analysis and Statistics

*R Studio* was used for downstream analyses. The *tidyverse* set of packages was used for data manipulation and visualisations, in particular *ggplot2* (Wickham 2016; Wickham et al. 2019). Dotplots show mean ± c.i (package *ggpubr* (Kassambara 2023a)). Statistical comparisons were carried out using the package *rstatix* (Kassambara 2023b). *Pheatmap* and *ComplexHeatmap* were used to generate Pearson correlation heatmaps (Gu et al. 2016; Kolde 2019).

### 4.8 Diversity Indices

TCRs or CDR3s were subsampled (rarefied) to a number lower than the smallest repertoire being compared using the package *vegan* (Oksanen et al. 2022) to correct for differences due to sample size. Mean diversity indices were calculated from 1000 repeats of this random sampling. In the steady‐state thymus, for CD3^−/lo^DP 500 TCRs were sampled 1000 times; and for CD3^+/hi^DP, SP4 and SP8 2000 TCRs were sampled 1000 times. In the HC‐treated thymus, for CD3^−/lo^DP repertoires 750 TCRs were sampled 1000 times; and for CD3^+/hi^DP 3000 TCRs were sampled 1000 times before calculation of diversity indices. Given the difference in sample size used for analyses between CD3^−/lo^DP repertoires and all other repertoires, diversity indices cannot be directly compared for CD3^−/lo^DP and other repertoires, so diversity indices for CD3^−/lo^DP repertoires were plotted separately. Shannon Entropy was computed using the package *vegan* (Oksanen et al. 2022); Gini index using *ineq* package (Zeileis and Kleiber 2014); Jaccard Index of Similarity using the base R function *dist*.

### 4.9 Principal Component Analysis

Before PCA was applied, raw counts were log_10_ transformed to account for differences in sample size, using a pseudocount of 0.01 for any counts that were not observed. Z‐scores were calculated from these values by subtracting the mean and dividing by the standard deviation. PCA was computed using the base R function *prcomp*, and the *factoextra* package was used to investigate the results (Kassambara and Mundt 2020).

### 4.10 VxJ Combinations

Proportional VxJ gene usage of TCR was calculated for each sample, natural log transformed and only VxJ combinations that were detected in at least three samples from each life‐stage were compared. Statistical comparisons were carried out by unpaired Student's *t*‐test followed by FDR‐adjustment (5%, Benjamini‐Hochberg procedure) of *p* values.

### 4.11 Sequential Amino Acid Triplet Analysis (k‐mer Analysis)

CDR3 sequences can be broken down into small amino acid motifs made up of sequential amino acid triplets (k‐mers), which can provide insight into the specificity of the TCR (Thomas et al. 2013). We calculated the distribution of all possible combinations of any three sequential amino acids (3‐mers) found within the CDR3 region of each TCR from each repertoire. These conserved sequential amino acid triplets (k‐mers) were extracted from CDR3 sequences using the *immunarch* package (ImmunoMind Team 2019).

## Author Contributions

J.R., B.C. and T.C. conceived the study; J.R. and T.C. designed experiments and wrote the manuscript. J.R. and O.A.P. analysed sequencing data; J.R., D.Y., E.Z., S.R., A.M. and C.‐I.L. performed experiments. B.C. critically reviewed the manuscript.

## Funding

This work was supported by the Medical Research Council (MR/S037764/1) and Biotechnology and Biological Sciences Research Council (BB/Y010175/1, BB/T020970/1, BB/Y003454/1) to TC. J.R. was supported by a studentship from the BBSRC London Interdisciplinary Biosciences Consortium (1903458). Research at UCL Great Ormond Street Institute of Child Health is supported by the NIHR Biomedical Research Centre at Great Ormond Street Hospital and UCL.

## Conflicts of Interest

The authors declare no conflicts of interest.

## Supporting information


**Figure S1:** Age‐related changes to thymocyte subset distribution determined.
**Figure S2:** Age‐related changes in the diversity and distribution of the thymic.
**Figure S3:** Hydrocortisone treatment of 4‐week and 12‐month mice.
**Table S1:** Antibodies used in flow cytometry analysis and sorting.

## Data Availability

TCR sequencing data is available on UCL Research Data Repository at https://doi.org/10.5522/04/29291846.
