# Supplementary material for: The Thymic Microenvironment Shapes Age‐Related Qualitative Changes in the TCR Repertoire
Source: Aging Cell. 2026 Aug 20;25(9):e70675. doi: 10.1111/acel.70675 (PMC13494363; doi:10.1111/acel.70675)
Supplement: Supplementary file 1 [file ACEL-25-e70675-s001.zip › acel70675-sup-0004-FigureS1-S3-TableS1@Supplementary Figures and Table 030826.pdf]

Supplementary Figures and Table

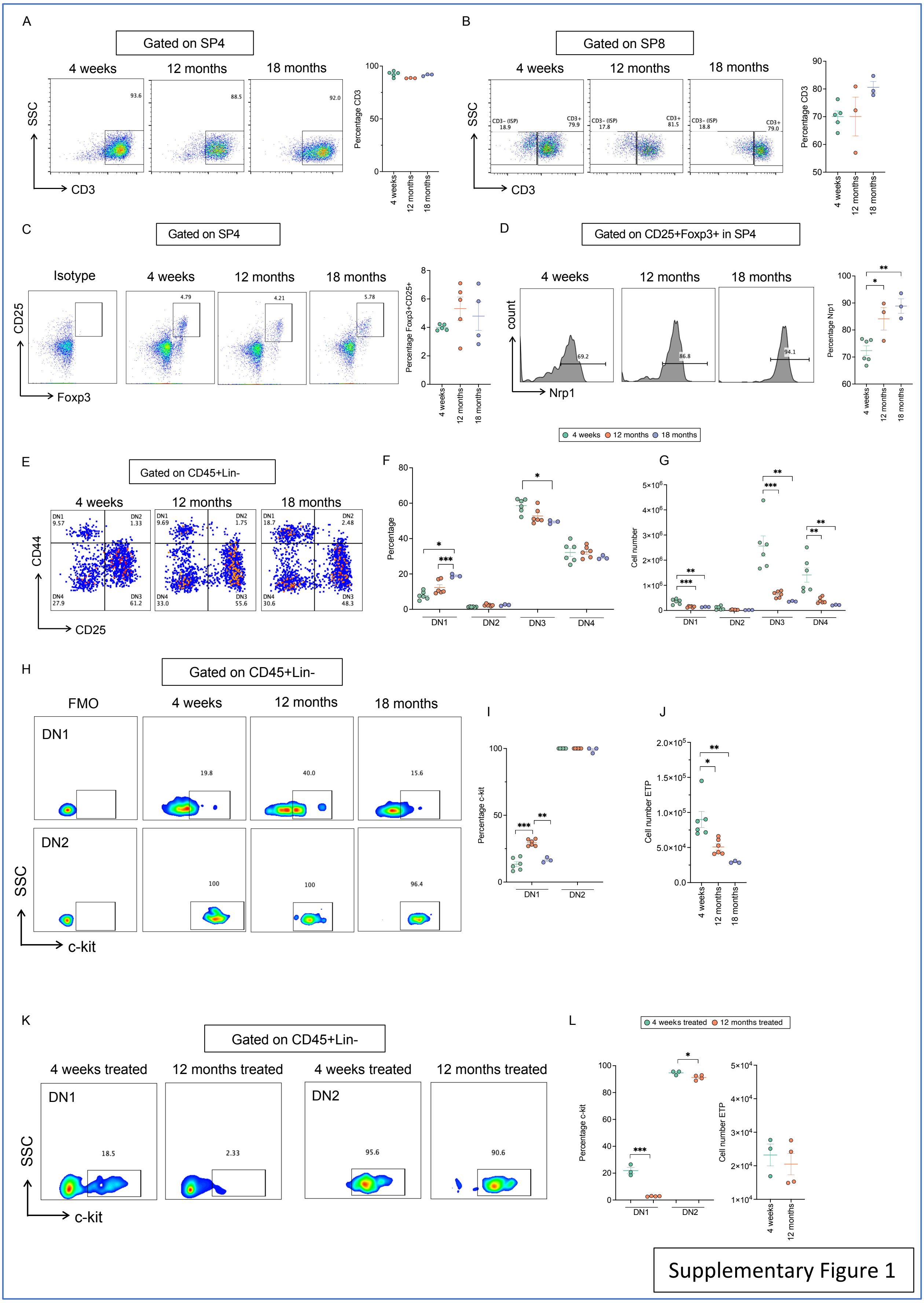

Supplementary Figure 1. Age-related changes to thymocyte subset distribution determined by flow cytometry

(A-B) Flow cytometry plots (left) show expression of CD3 in 4-week, 12-month and 18-month thymus (n=3-5), gated on **(A)** SP4; and **(B**) SP8. Dot plots (right) show percentage of CD3+ cells in these populations at the different ages (mean ± SEM). Statistical comparisons were carried out by one-way ANOVA (significant *p* values shown). **(C)** Flow cytometry plots (left) show expression of CD25 and intracellular (ic) Foxp3 gated on SP4 from 4-week, 12-month and 18-month thymus (n=4-5). Dotplot (right) shows percentage of Foxp3+CD25+ in CD4SP in each group, where each symbol represents an individual mouse, and line represents the mean. **(D)** Histograms show expression of Nrp1 gated on CD25+Foxp3+ SP4 (Treg). Dot plots show percentage of Nrp1 in each group (n=3-6). Error bars ± SEM. Statistical comparisons were carried out by one-way ANOVA (significant *p* values shown **$p < 0.01$, *$p < 0.05$). **(E)** Dot plots show expression of CD44 and CD25 markers gated on CD45+Lin-(CD3-CD4-CD8-CD11b-NK1,1-Ter119-CD19-) to identify DN populations at 4-weeks (n=6), 12-months (n=6) and 18-months (n=3). **(F-G)** Dot plots show percentage **(F)** and cell number **(G)** of DN cell populations (mean ± SEM). **(H)** Flow cytometry plots show c-kit expression in DN1 and DN2 populations gated on CD45+Lin- populations at 4-weeks, 12-months and 18-months and FMO as negative control for c-kit. **(I)** Dot plots show the percentage of c-kit in DN1 and DN2 populations. **(J)** Dot plots show cell number of ETP (ckit+) in CD45+Lin-DN1 and DN2 populations. Each point represents a thymus from an individual mouse (mean ± SEM). **(K)** Flow cytometry plots show the percentage of c-kit in DN1 and DN2 populations after 3 days of hydrocortisone treatment from 4-weeks (n=3) and 12-months old mice (n=4). **(L)** Dot plots show the percentage (left) of c-kit in DN1 and DN2 populations and (right) cell number of ETP (ckit+) in CD45+Lin-DN1/DN2 populations after 3 days of hydrocortisone treatment. Each point represents a thymus from an individual mouse (mean ± SEM). Statistical comparisons were performed within each cell-type and were carried out by unpaired Student's t-test: *** $p < 0.001$; ** $p < 0.01$; *$p < 0.05$.

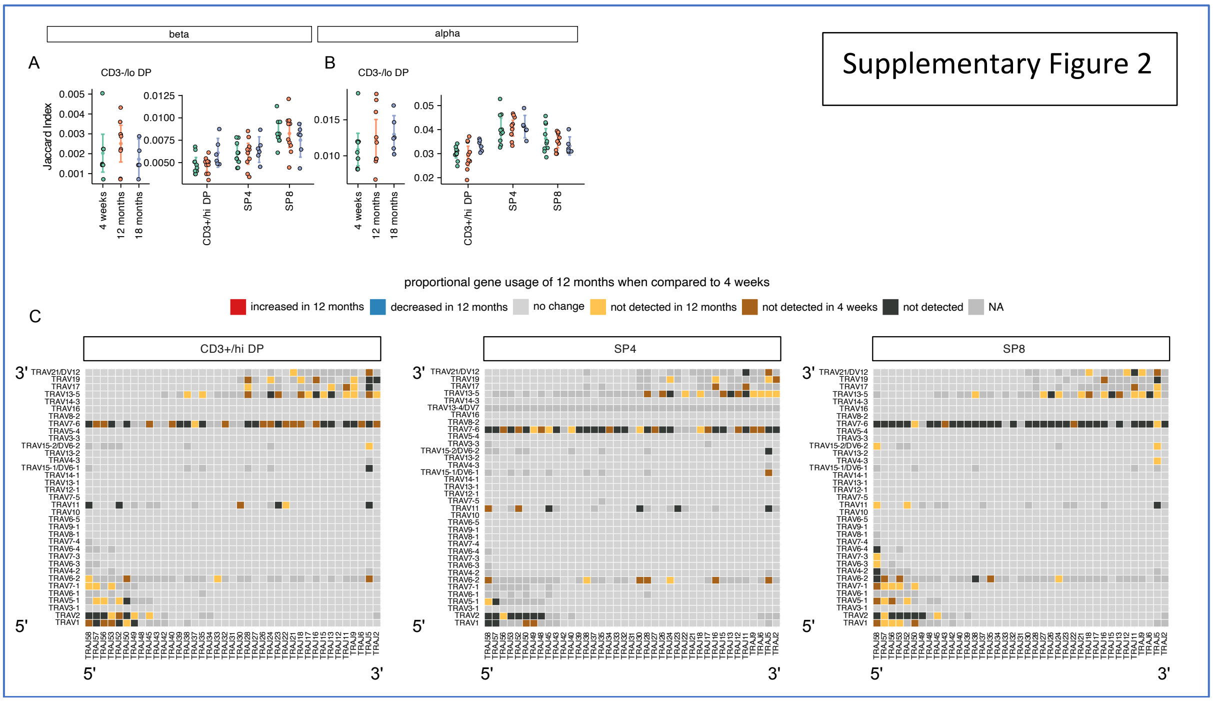

Supplementary Figure 2. Age-related changes in the diversity and distribution of the thymic TCRa repertoire

TCR α-chain repertoires were sequenced from FACS-sorted CD3$^{-/lo}$CD4^{+}CD8^{+} (CD3$^{-/lo}$DP), CD3$^{+/hi}$CD4^{+}CD8^{+} (CD3$^{+/hi}$DP), CD4^{+}CD8^{-}CD3^{+} (SP4) and CD4^{-}CD8^{+}CD3^{+} (SP8) thymocyte populations from 4-week (green circles; n=5), 12-month (orange circles; n=5) and 18-month-old (purple circles; n=4) C57BL/6 thymus.

(A-B) The intra life-stage Jaccard Index of Similarity of β-chain **(A)** and α-chain **(B)** CDR3s for 4-weeks, 12-months and 18-months in the CD3$^{-/lo}$DP (left) and CD3$^{+/hi}$DP, SP4 and SP8 populations (right). Each repertoire was subsampled to 500 (CD3$^{-/lo}$DP) or 1500 (CD3$^{+/hi}$DP, SP4 and SP8) CDR3s 1000 times before calculating the Jaccard Index. **(C)** Plots show comparison between α-chain log normalised VxJ gene usage of unique TCRs for 12-months compared to 4-weeks in CD3$^{+/hi}$DP, SP4 and SP8 populations. Red tiles signify increased usage of VxJ combinations in 12-months ($p < 0.05$), while blue tiles signify decreased usage in 12-months ($p < 0.05$) compared to 4-weeks. Light grey tiles signify no change in gene usage ($p > 0.05$). Only combinations that were detected in at least 3 mice per group were compared. Statistical comparisons were carried out by unpaired Student's t-test followed by FDR-adjustment (5%, Benjamini-Hochberg procedure) of *p* values.

Plots show mean±c.i. Statistical comparisons were performed within each cell-type and were carried out by one-way ANOVA followed by Tukey post-hoc tests (significant *p* values shown) *** p <0.001; ** p <0.01; *p < 0.05.

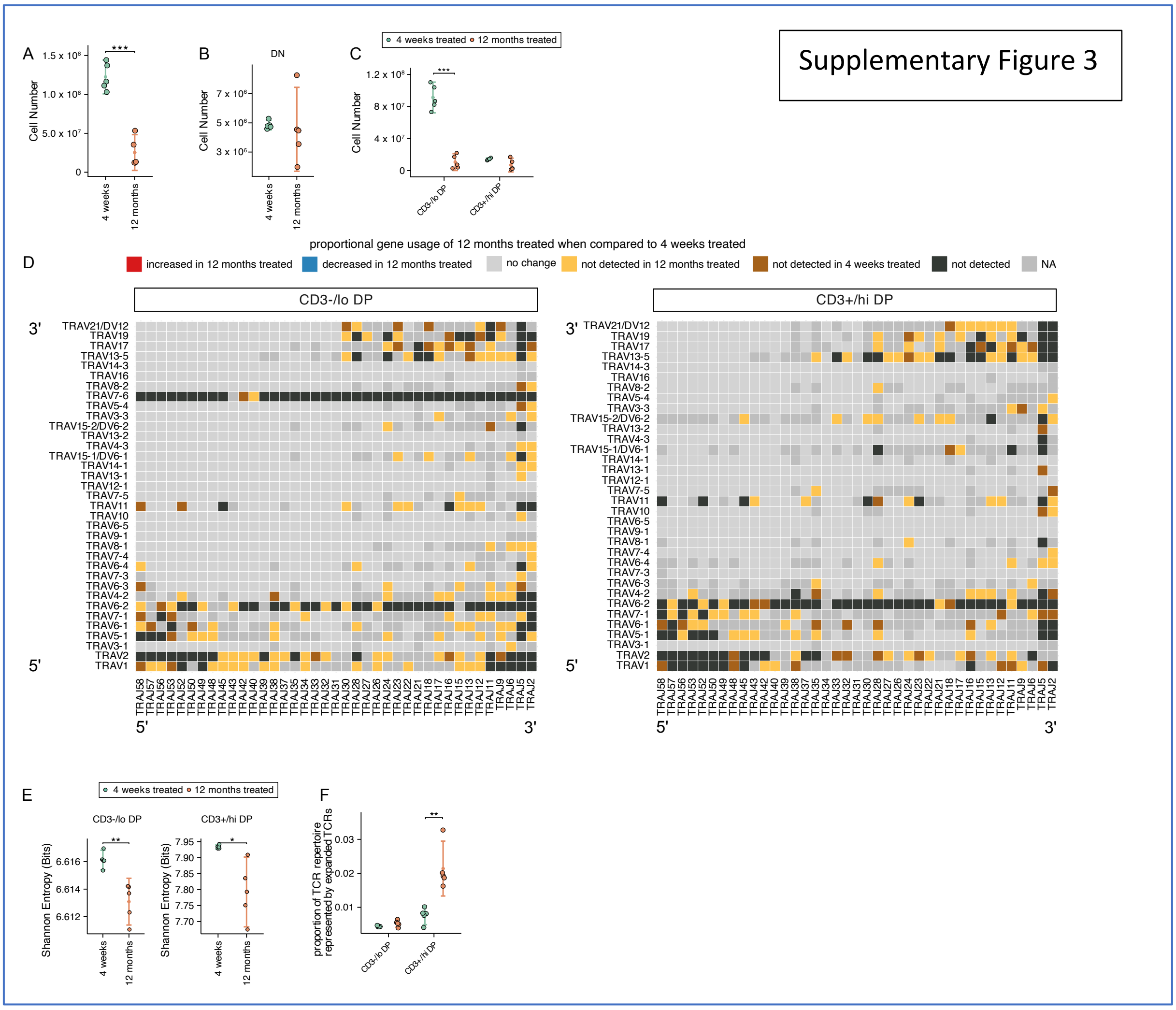

Supplementary Figure 3. Hydrocortisone treatment of 4-week and 12-month mice

Thymus was analysed on day 6 after hydrocortisone treatment. TCR a-chain repertoires were sequenced from FACS-sorted $CD3^{-/lo}CD4^{+}CD8^{+}$ ($CD3^{-/lo}DP$) and $CD3^{+/hi}CD4^{+}CD8^{+}$ ($CD3^{+/hi}DP$) thymocyte populations from 4-week-old treated with hydrocortisone on day 6 **(D-F)** after treatment; 4-week treated (green circles; n=5) and 12-month-old treated (orange circles; n=5) C57BL/6 thymus.

(A) The number of cells recovered from HC-treated 4-week and 12-month thymus on day 6, where each point represents an individual mouse. **(B)** The number of DN cells recovered from 4-week and 12-month HC-treated thymus on day 6, where each point represents an individual mouse. **(C)** The number of $CD3^{-/lo}DP$ and $CD3^{+/hi}DP$ cells recovered from 4-week and 12-month HC-treated thymus on day 6, where each point represents an individual mouse. **(D)** Plots show comparison between α-chain log normalised VxJ gene usage of total TCRs for 12-months treated compared to 4-weeks-treated in $CD3^{-/lo}DP$ and $CD3^{+/hi}DP$ populations. Red tiles signify increased usage of VxJ combinations in 12-months-treated ($p < 0.05$), while blue tiles signify decreased usage in 12-months treated ($p < 0.05$) compared to 4-weeks-treated. Light grey tiles signify no change in gene usage ($p > 0.05$), yellow tiles signify VxJ combinations not detected in 12-months-treated, brown tiles signify VxJ combinations not detected in 4-weeks-treated and black tiles signify VxJ combinations not detected in both 12-months-treated and 4-weeks-treated. Dark grey tiles signify combinations that were not compared. Statistical

comparisons were carried out by unpaired Student's t-test followed by FDR-adjustment (5%, Benjamini-Hochberg procedure) of *p* values. Only combinations that were detected in at least 3 mice per group were compared. **(E)** The Shannon entropies for the α-chain TCR repertoires of 4-weeks-treated and 12-months-treated in $CD3^{-/lo}$DP (left plot) and $CD3^{+/hi}$DP (right plot) populations, where each point represents an individual mouse. Each repertoire was subsampled to 750 ($CD3^{-/lo}$DP) or 3000 ($CD3^{+/hi}$DP) TCRs 1000 times before calculating the Shannon entropy. **(F)** The proportion of the TCR α-chain repertoire accounted for by the expanded top 1% most abundant sequences (>99th percentile) for 4-weeks-treated and 12-months-treated in $CD3^{-/lo}$DP and $CD3^{+/hi}$DP populations, where each point represents an individual mouse.

Dotplots show mean$\pm$c.i. Statistical comparisons were performed within each cell-type and were carried out by unpaired Student's t-test or Welch's t-test as appropriate: *** $p < 0.001$; ** $p < 0.01$; *$p < 0.05$.

Supplementary Table 1: Antibodies used in flow cytometry analysis and sorting

Antibody	Fluorochrome	Clone
CD4	APC	RM4-4
CD4	PerCP-Cy5.5	RM4-4
CD4	FITC	RM4-4
CD4	PE	RM4-4
CD8	BV421	53.6.7
CD8	BV605	53.6.7
CD8	FITC	53.6.7
CD8	PE/Cyanine7	53.6.7
CD8	PE	53.6.7
CD62L	APC	MEL-14
TCRb	PE/Cyanine7	H57-597
CD44	BV421	IM7
CD44	PerCP/Cyanine5.5	IM7
CD25	APC	3C7
CD25	FITC	3C7
CD25	PE	3C7
CD3	PerCP-Cy5.5	17-A2
CD3	PE	17-A2
CD3	FITC	17-A2
CD71	PE	R17217
CD71	FITC	R17217
Foxp3	APC	236A/E7
FOXP3	APC	FJK-16s
Nrp1	PE/Cyanine7	3E12
NK1.1	AF700	PK136
NK1.1	PE	PK136
c-kit	APC	2B8
c-kit	BV421	2B8
CD45	PE/Cyanine7	S18009F
CD45	APC/Cyanine7	S18009F
CD11b	BV711	M1/70

CD11b	PE	M1/70
Ter119	PerCP/Cyanine5.5	Ter119
Ter119	PE	Ter119
CD19	PE	6D5
CD19	APC/Cyanine7	6D5
