# Supplementary material for: The Thymic Microenvironment Shapes Age‐Related Qualitative Changes in the TCR Repertoire
Source: Aging Cell. 2026 Aug 20;25(9):e70675. doi: 10.1111/acel.70675 (PMC13494363; doi:10.1111/acel.70675)
Supplement: Supplementary file 1 [file ACEL-25-e70675-s001.zip › acel70675-sup-0001-FigureS1@Supplementary Figure 1 thymus-2.pdf]

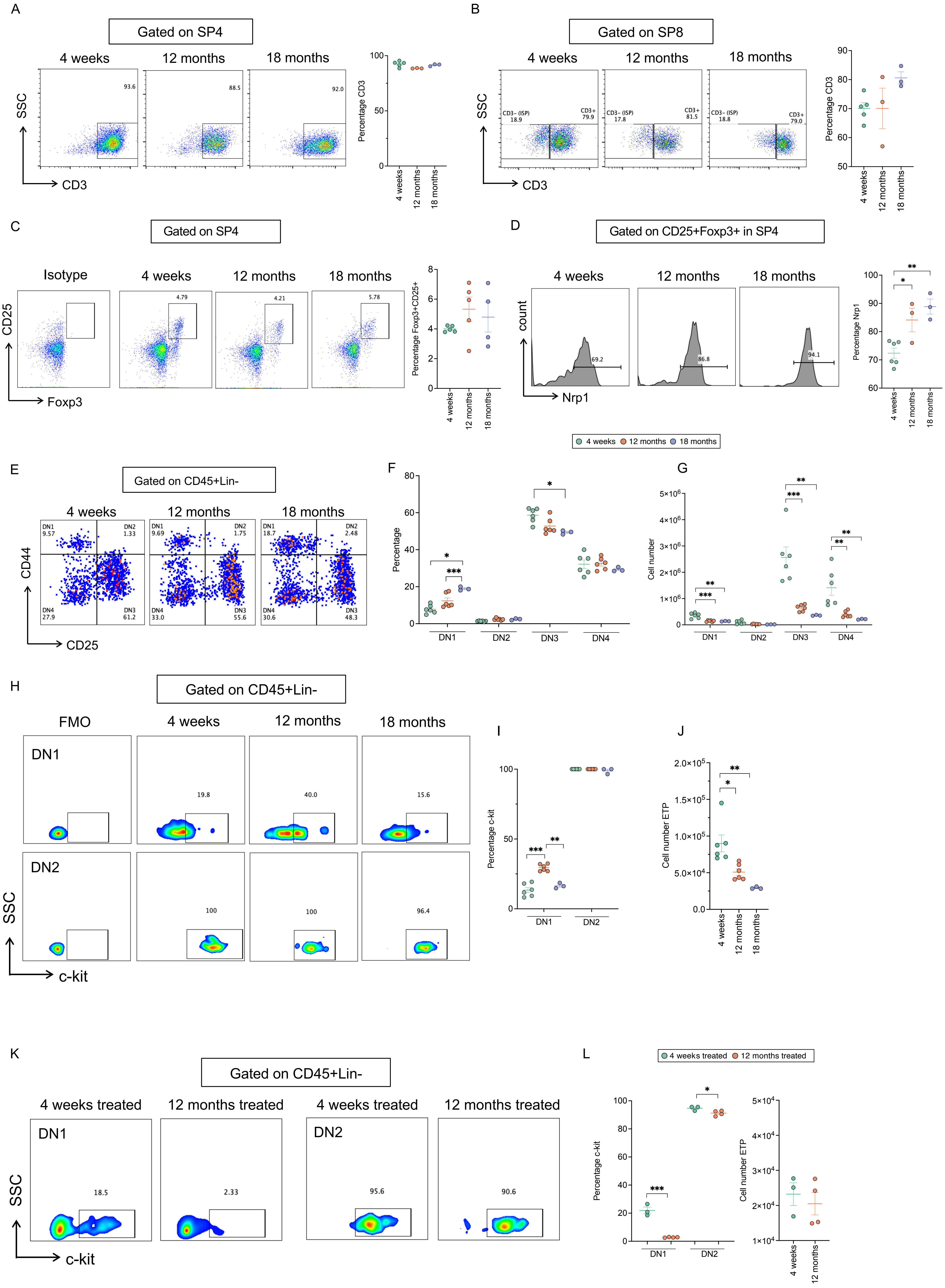

Supplementary Figure 1
